# Landscape genomics of the streamside salamander: Implications for species management in the face of environmental change

**DOI:** 10.1111/eva.13321

**Published:** 2022-01-25

**Authors:** Marc A. Beer, Rachael A. Kane, Steven J. Micheletti, Christopher P. Kozakiewicz, Andrew Storfer

**Affiliations:** ^1^ School of Biological Sciences Washington State University Pullman Washington USA; ^2^ Department of Microbiology, Immunology, and Pathology Colorado State University Fort Collins Colorado USA

**Keywords:** adaptation, climate change, conservation genetics, population genetics—empirical

## Abstract

Understanding spatial patterns of genetic differentiation and local adaptation is critical in a period of rapid environmental change. Climate change and anthropogenic development have led to population declines and shifting geographic distributions in numerous species. The streamside salamander, *Ambystoma barbouri*, is an endemic amphibian with a small geographic range that predominantly inhabits small, ephemeral streams. As *A*. *barbouri* is listed as near‐threatened by the IUCN, we describe range‐wide patterns of genetic differentiation and adaptation to assess the species’ potential to respond to environmental change. We use outlier scans and genetic‐environment association analyses to identify genomic variation putatively underlying local adaptation across the species’ geographic range. We find evidence for adaptation with a polygenic architecture and a set of candidate SNPs that identify genes putatively contributing to local adaptation. Our results build on earlier work that suggests that some *A. barbouri* populations are locally adapted despite evidence for asymmetric gene flow between the range core and periphery. Taken together, the body of work describing the evolutionary genetics of range limits in *A. barbouri* suggests that the species may be unlikely to respond naturally to environmental challenges through a range shift or *in situ* adaptation. We suggest that management efforts such as assisted migration may be necessary in future.

## 1 INTRODUCTION

A central goal of molecular ecology and evolutionary biology is to understand the genetic basis of local adaptation (Hoban et al., 2016; Storz, 2005). Landscape genomics has emerged as an analytical framework to achieve this goal (e.g., Joost et al., 2007; Manel & Holderegger, 2013; Storfer et al., 2018), which is particularly pertinent in light of the potential influence of climate change on species’ geographic distributions (Razgour et al., 2019). Indeed, many species are already shifting their geographic distributions toward the poles (Chen et al., 2011; Hickling et al., 2006; Parmesan et al., 1999; Parmesan & Yohe, 2003; Pecl et al., 2017), and approximately a quarter of species assessed by the IUCN face the threat of extinction (IUCN, 2020). Climate change is likely to have disproportionate impacts on endemic species with small geographic distributions because such species tend to have narrow tolerance ranges to climatic factors such as temperature and precipitation (Sheth & Angert, 2014; Urban, 2015; Yu et al., 2017).

Species range limits often exist, in theory, because populations at the range edge are small and lack genetic diversity to adapt beyond the edge (Eckert et al., 2008; Sexton et al., 2009). Edge populations are thus often the first to disappear when a species undergoes a range‐wide decline (Doherty et al., 2003). Landscape genomics studies elucidate patterns of adaptive genomic diversity that management initiatives can use to conserve the capacity of taxa to respond to environmental change (Funk et al., 2019). For example, assisted migration has emerged as one strategy to relocate populations of species threatened by climate change to areas of suitable habitat (Hällfors et al., 2014; Vitt et al., 2010). Another strategy is genetic rescue, or the process of introducing individuals from more genetically variable populations into those that are genetically depauperate to enhance their adaptive potential and fitness (Fitzpatrick et al., 2020; Pimm et al., 2006; Whiteley et al., 2015). When specific threats are known (e.g., increasing average temperatures), conservation efforts may benefit from genotyping individuals and translocating those identified as harboring putatively beneficial alleles (Hohenlohe et al., 2019).

Amphibians appear particularly sensitive to threats imposed by climate change (Blaustein et al., 2010; Lourenço‐de‐Moraes et al., 2019), owing to their limited dispersal abilities to track climate shifts and frequent reliance on ephemeral habitats for breeding and larval survival (Blaustein et al., 2010). The streamside salamander, *Ambystoma barbouri* (Kraus & Petranka, 1989), is an endemic species with a small geographic range primarily restricted to central Kentucky, southeastern Indiana, and southwestern Ohio (Kraus & Petranka, 1989; Petranka, 1998). *A. barbouri* is listed as near‐threatened by the IUCN, which cites urban and agricultural development, forest harvesting, invasive species, and climate change as drivers (Hammerson, 2004). *A. barbouri* adults lay eggs with consequent larval development primarily in small, ephemeral streams that are prone to early drying in hot years (Petranka, 1998). Larval activity varies with stream permanence, and experiments suggest a strong genetic component that limits the extent of phenotypic plasticity of developmental timing, which may experience selection (Micheletti & Storfer, 2020; Storfer & Sih, 1998).

On a range‐wide scale, *A. barbouri* conforms to the expectations of the central‐marginal hypothesis (Brown, 1984; Eckert et al., 2008). That is, there is a negative correlation of genetic diversity, effective population size, and population connectivity moving from the core of the species’ distribution to the edge (Micheletti & Storfer, 2015). Additionally, despite asymmetric gene flow along transects from the range core to range periphery, a reciprocal transplant field experiment suggests that edge populations show evidence of local adaptation (Micheletti & Storfer, 2020). The geographic range of *A. barbouri* appears to be restricted owing to a combination of relatively cryptic environmental variables elucidated by a landscape genetics study, including limited limestone availability and increases in growing season precipitation that create high resistance to gene flow (Micheletti & Storfer, 2017). To date, however, genetics studies on *A. barbouri* have been conducted on small numbers of presumably neutral loci, including allozymes (Storfer, 1999) and microsatellites (Micheletti & Storfer, 2015, 2017). Collectively, the relatively strong genetic isolation among study sites (Storfer, 1999) and evidence for local adaptation at a range‐wide scale warrants further investigation of the genomic basis of adaptation and the capacity to respond to environmental change in this species.

**FIGURE 1:**
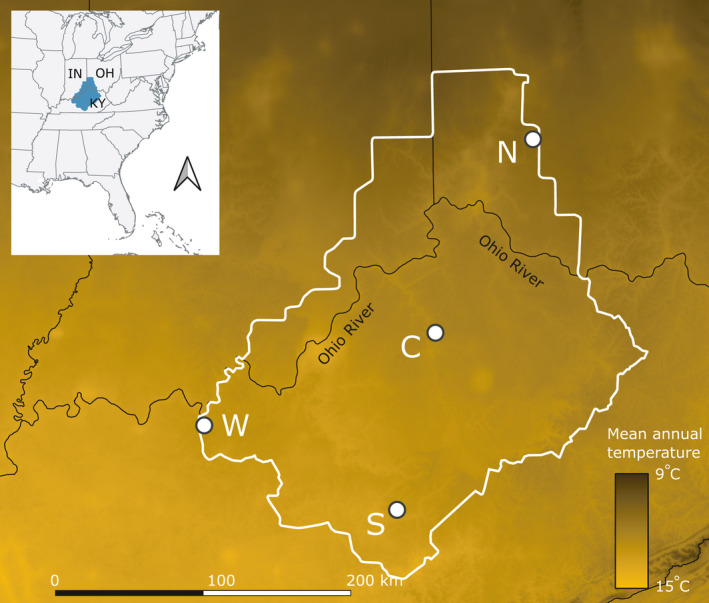
Map indicating the four collection localities. The continuous geographic range of *Ambystoma barbouri* is indicated as the white outline in the main plot and blue shaded region in the inset. Tissue samples of 28 larval *A. barbouri* were collected from each of four locations, marked with white points and location labels, corresponding to the range center (C) and the extreme northern, western, and southern range edges (N, W, and S, respectively). Map features are plotted over mean annual temperature

The major goal of this study was to identify genetic markers showing large signatures of divergent selection among study sites spanning the geographic range of *A. barbouri*, including a site at the core, as well as sites at the northern, southern, and western range edges (Figure 1). Specifically, we used double‐digest restriction site‐associated DNA (ddRAD) sequencing (Peterson et al., 2012) to: (1) describe genetic differentiation among range core and peripheral study sites; (2) evaluate genomic evidence for local adaptation; and (3) gain insight into the potential for adaptive responses to environmental change.

## 2 MATERIALS AND METHODS

### 2.1 Field collection

We collected 112 tissue samples across four collection localities each separated by an average of 190.62 km within the continuous distribution of *A. barbouri* between April and May 2016 (Figure 1). The collection localities included fishless, ephemeral streams in the geographic core of the distribution and at the extreme northern, southern, and western edges. We selected these specific edge sites because previous research suggests that they belong to genetically differentiated clusters (Micheletti & Storfer, 2017) and would allow the characterization of range‐wide genetic variation. The tissue samples from each site consisted of tail tips from larval salamanders, which were collected among different stream pools within single stream basins to avoid sampling full siblings. Tissues were stored in 95% ethanol.

### 2.2 Next‐generation sequencing

We performed double‐digest restriction site‐associated DNA sequencing (ddRADseq; Peterson et al., 2012), using the restriction enzymes EcoRI and PstI, both of which use six‐base‐pair recognition sites. We multiplexed individuals with 30 unique adapter barcodes. We size‐selected 500–600 bp fragments using a Pippin Prep Blue (Sage Science) and amplified final libraries using Phusion PCR master mix (Thermo Fisher Scientific). Due to the large genome size of *A. barbouri* (approximately 24 Gb; www.genomesize.com), we included only 30 individuals in each of four final libraries to attempt to increase sequencing depth across individuals; two individuals from each sampling location were duplicately sequenced in different libraries. A total of four libraries were sequenced on an Illumina HiSeq 2000 sequencing system at the University of Oregon Genomics Core Facility (gc3f.uoregon.edu) using single‐end, 100 bp reads.

### 2.3 Variant calling and filtering

We carried out an integrated alignment procedure (Paris et al., 2017) by first assembling RAD loci *de novo* in Stacks Version 2.52 (Rochette et al., 2019) and then using the *mem* option in BWA (Li & Durbin, 2009) to align catalogued *de novo* loci to the June 2018 chromosome‐level genome assembly of *A. mexicanum* (Smith et al., 2019), the most closely related salamander genome available. The de novo RAD locus catalog was created using the Stacks script *denovo_map*.*pl*, with options set to require a minimum of three reads to form a stack (*m* = 3), allow three mismatches between read stacks within individuals (*M* = 3), and allow four mismatches between read stacks across individuals (*n* = 4). These parameter values were optimized by following Paris et al. (2017). For alignment, the clipping penalty for BWA‐mem was specified as 10 and all other settings were left as default. Mapping information for RAD loci with MAPQ > 10 was integrated with the Stacks RAD locus catalog using the Stacks script *stacks*‐*integrate*‐*alignments*.*pl*.

We filtered the resulting single nucleotide polymorphisms (SNPs) using an iterative filtering procedure modified from O’Leary et al. (2018). Briefly, iterative filtering involves alternating and progressively increasing the stringency of filters, a process that may generate a final dataset containing a larger number of retained individuals and higher‐quality markers than a filtering process that applies each filter at a strict level only once (O’Leary et al., 2018). The iterative filtering stage of our pipeline involved cycling between removing SNPs with high missingness in at least one of the four collection localities and removing individuals with high missingness. We also highlight that we employed a number of filters intended to reduce the abundance of RAD loci representing collapsed paralogs, which may otherwise be prevalent given the large, repetitive genomes of salamanders. The full filtering scheme is detailed in Table S1.

We evaluated the genomic distribution of SNPs in the final dataset by correlating numbers of SNPs that mapped to each reference chromosome with reference chromosome size; a strong correlation would suggest proportional representation of chromosomes in our dataset. We also characterized whether SNPs were within or between gene models of the *A. mexicanum* genome. We first downloaded BED files containing gene model annotations for each chromosome of the June 2018 *A. mexicanum* genome assembly from the Table Browser of the UCSC Genome Browser (www.genome.ucsc.edu) and then used the BEDTools *closest* command (Quinlan & Hall, 2010) to identify the gene models nearest each SNP.

### 2.4 Summary statistics and population genetic structure

We used fastStructure (Raj et al., 2014) and the *find*.*clusters* function in the R package Adegenet 2.1.3 (Jombart, 2008) to infer the number of genetic clusters represented in the dataset. For fastStructure, we assessed support for *K* = 1–10 using 20 replicates for each value of *K* and subsequently used the python script *chooseK* included with fastStructure to identify the value of *K* with the greatest statistical support. We visualized admixture proportions of individuals using the software Distruct v1.1 (Rosenberg, 2004). The Adegenet *find*.*clusters* function transforms data using principal components analysis (PCA) before using a successive *K*‐means clustering algorithm to evaluate support for different values of *K*. We assessed results for *K* = 1–10 and selected the value of *K* with the lowest value of the Bayesian Information Criterion. We also employed PCA and discriminant analysis of principal components (DAPC) in Adegenet (Jombart, 2008). Prior to performing PCA and DAPC, we used the *impute* function from the R package LEA (Frichot & François, 2015) to impute missing data based on ancestry and genotype frequency estimates obtained from the best run of 500 replicates of the *snmf* function. We used xval optimization to determine the number of principal components to be used in DAPC and retained *K* discriminant axes. We evaluated both PC and DAPC scores of individuals because PCA characterizes variation occurring both within and between genetic groups, whereas DAPC focuses on variation between groups (Jombart et al., 2010). We additionally evaluated the correlation between PCs, latitude, and longitude using Pearson's correlation test (e.g., Novembre et al., 2008).

We used the R package diveRsity (Keenan et al., 2013) to calculate Weir and Cockerham's estimators of the inbreeding coefficient *F*_IS_ and *F*_ST_ (Weir & Cockerham, 1984). We estimated *F*_IS_ for each population using the *basicStats* function and estimated 95% confidence intervals using 1000 bootstraps. We estimated 95% confidence intervals (CI) for pairwise *F*_ST_ based on 1000 bootstraps using the bias‐corrected bootstrapping method implemented in the *diffCalc* function; we carried out bootstrapping across SNPs, as recommended by Weir and Cockerham (1984). We also recalculated pairwise *F*_ST_ after removing SNPs determined to have significant signatures of selection in univariate tests (see below). We further characterized genetic diversity for each locality by calculating individual observed heterozygosity and per‐SNP nucleotide diversity (*π*) in VCFtools (Danecek et al., 2011). We estimated effective population size (*N*_e_) for each locality using the linkage disequilibrium‐based method in NeEstimator v2.1 (Do et al., 2014); *N*_e_ estimates were obtained after removing SNPs with significant signatures of selection. We repeated effective population size estimates using allele frequency thresholds of 0.05, 0.02, and 0.01.

### 2.5 Environmental variables

We initially downloaded GIS layers for the 19 bioclimatic variables available from the WorldClim database (Fick & Hijmans, 2017) and six additional variables, briefly outlined here (Table S2). The bioclimatic variables are all related to temperature and precipitation, which are related to known stressors in amphibians (e.g., desiccation; Daszak et al., 2005). Temperature seasonality may select for the width of thermal tolerance (i.e., the difference between upper and lower thermal limits) in amphibians (Snyder & Weathers, 1975). The bioclimatic variables also include quarter‐specific environmental conditions, which appear to impact habitat suitability for *A. barbouri* (Micheletti & Storfer, 2015).

We also included solar radiation averaged across months and elevation in our analyses (Fick & Hijmans, 2017) because ultraviolet radiation exposure negatively correlates with aspects of fitness in various amphibian species (Bancroft et al., 2008). *A. barbouri* also favors forested habitat (Kraus & Petranka, 1989), so we included percent forest canopy cover (Coulston et al., 2012) and land cover (Homer et al., 2020) as additional variables. Land cover was transformed into a binary variable of forest habitat (any of three habitat types: deciduous, conifer, and mixed forest) versus nonforest habitat. Adult *A. barbouri* are fossorial, spending considerable time underground, suggesting that soil chemistry may impact fitness. Soil pH and percent soil organic carbon both correlate with many biophysicochemical properties of soil (Brady & Weil, 1999; Smith & Doran, 1997), so we included both factors, predicted for a depth of 5 cm, as additional variables (Ramcharan et al., 2018). We converted pH to the corresponding concentration of hydronium ions prior to analyses.

Using QGIS Desktop 3.12.1 (QGIS development team, 2009), we defined a 2.5 km radius buffer zone around each collection locality; the larvae sampled are unlikely to be offspring of individuals that dispersed from locations outside of this buffer (e.g., Orloff, 2011; Rittenhouse & Semlitsch, 2007). We then extracted the mean value of each environmental variable within the buffer to determine broad differences in environmental conditions among sites. For the binary variable of forest habitat vs. nonforest habitat, we calculated the proportion of the buffer zone corresponding to forest habitat. The full set of variables and values for each collection locality can be found in Table S2. Note that the geographic range of *A. barbouri* is small, so the species’ environmental tolerance ranges are likely narrow (Micheletti & Storfer, 2015; Sheth & Angert, 2014; Yu et al., 2017). Although environmental factors may appear to have little variation among collection localities, this variation may be important from a biological perspective. For example, the maximum and minimum elevations (differing by 110 m) observed among our sites are similar to those identified from species presence data used in Micheletti and Storfer (2015).

For genetic‐environment association (GEA) tests, we reduced the number of highly correlated environmental variables using a two‐stage approach. In the first stage, we grouped logically related variables (e.g., temperature‐related variables) and retained only those variables correlated at *r* ≤ 0.90 within each group. We then pooled the remaining variables and again retained only those correlated at *r* ≤ 0.90. A matrix of Pearson's correlation coefficients for every pair of variables can be found in Table S3. Although this process filtered out annual precipitation, we retained this variable because it is relevant for explaining the distribution of *A. barbouri* (Micheletti & Storfer, 2017) and generally relevant to various aspects of amphibian ecology (e.g., aquatic breeding, aquatic larval stage, and vulnerability to desiccation); imperfectly correlated variables also may reveal nonredundant signatures of selection. This yielded a final set of six variables: mean annual temperature, temperature seasonality, mean temperature of the wettest quarter, annual precipitation, elevation, and percent soil organic carbon (Table 1). Using these six variables, we additionally quantified environmental distance among the sampling locations. Environmental variables were demeaned and divided by their standard deviations; we conducted PCA on the resulting centered and scaled environmental data and then calculated pairwise Euclidean distances among the four sites using all principal components (e.g., Wang, 2013). We also assessed correlations among pairwise environmental distance, geographic distance, and linearized *F*_ST_ (i.e., *F*_ST_/(1 − *F*_ST_); Rousset, 1997). We additionally assessed correlations between PC1 and PC2 from the genetic PCA (see above) and each of the six environmental factors independently.

**TABLE 1:** Final set of environmental factors used in genetic‐environment association (GEA) analyses

Sampling location	Sample size (final dataset)	Latitude	Longitude	Mean annual temperature (°C)	Temperature seasonality (°C)	Mean temperature of the wettest quarter (°C)	Elevation (m)	Soil organic carbon (%)	Annual precipitation (mm)
C	23	38.3227	−84.8319	12.49	8.89	21.07	192.19	2.79	1129.44
N	20	39.4861	−84.0277	11.1	9.23	20.09	276.93	3.03	1034.52
S	20	37.2468	−85.1561	13.27	8.4	21.4	260.19	3.38	1333.03
W	23	37.7765	−86.6265	13.12	8.89	12.9	167.46	2.60	1213.07

### 2.6 Tests for selection

We carried out several tests for selection that broadly belong to two categories: differentiation outlier scans and GEA analyses. Outlier scans identify loci showing unusual differentiation among populations relative to background genetic differentiation, while GEA analyses identify loci with allele frequencies that correlate significantly with environmental variables while accounting for neutral population structure (Hoban et al., 2016; Rellstab et al., 2015). We used stringent significance thresholds recommended by the authors of each test. When authors of tests did not clearly designate significance thresholds, we consulted the literature for common cutoffs.

We performed outlier detection analyses using the software hapflk v1.4 (Bonhomme et al., 2010), pcadapt (Luu et al., 2017), and the Bayenv2 X^T^X statistic (Günther & Coop, 2013). We used hapflk v1.4 to compute the FLK statistic for each SNP and assessed outlier significance at a false discovery rate threshold of q<0.05 (Benjamini & Hochberg, 1995). Pcadapt calculates *z*‐scores for each SNP by regressing each SNP against principal components; SNPs that emerge as outliers on the basis of Mahalanobis distances calculated from z‐scores are interpreted as putatively experiencing divergent selection among populations. We used the conservative Bonferroni correction of 0.01/10,527 (the number of SNPs) to assess the significance of each SNP (Luu et al., 2017). We additionally used the X^T^X statistic implemented in Bayenv2, which is a measure of subpopulation differentiation analogous to *F*_ST_. Bayenv2 accounts for neutral genetic structure using a population covariance matrix, which we obtained by calculating the median matrix across five replicate runs of Bayenv2 for 500,000 iterations each. Using the empirical covariance matrix, we then ran five independent replicates of Bayenv2 for 750,000 iterations and calculated the median value of X^T^X across replicates for each SNP. To calibrate the significance threshold of the X^T^X statistic, we used the BayPass accessory R function *simulate*.*baypass* to generate a pseudo‐observed dataset (POD) of neutral SNPs based on the empirical covariance matrix estimated in Bayenv2 (Gautier, 2015). The POD contained the same number of SNPs as the real dataset, and sample sizes for each SNP were drawn with replacement from the sample sizes in the real dataset. We calculated the X^T^X statistic for each SNP in the POD using Bayenv2, with options identical to the analysis carried out on the real dataset. We used the X^T^X value corresponding to the top 0.01% quantile of the POD as the threshold for significance in the real dataset (Gautier, 2015).

We implemented GEA tests using LFMM 2 (Caye et al., 2019) in the R package lfmm, as well as Bayenv2 (Günther & Coop, 2013). LFMM uses a number of latent factors (equal to the *K* inferred genetic clusters in the data) to account for neutral genetic structure when testing for associations between alleles and environmental factors. We accounted for multiple comparisons using a Bonferroni‐corrected significance threshold of 0.01/10,527 (Frichot et al., 2013; Yang et al., 2020). Bayenv2 calculates a Bayes factor (BF) for each locus‐environmental factor combination, which describes the support for a model which includes the environmental factor relative to a null model which excludes it. Bayenv2 also estimates Spearman's Rho, a rank‐based correlation coefficient describing the association between each SNP and environmental factor. Spearman's Rho is robust to outlier populations (Günther & Coop, 2013); Günther and Coop (2013) recommend the simultaneous use of a relatively permissive BF threshold and relatively stringent Spearman's Rho threshold when outlier populations are a concern. We ran five independent replicates of Bayenv2 for 750,000 iterations using the same covariance matrix used in the estimation of X^T^X and calculated the median Bayes factor and median absolute Spearman's Rho across replicates for each locus–environmental factor combination. We considered SNPs with both BFs in the top 5% quantile and absolute Spearman's Rho coefficients in the top 1% quantile as showing strong evidence for association with an environmental factor, which are thresholds consistent with other studies (e.g., Contreras‐Moreira et al., 2019).

We further characterized significant SNPs by evaluating overlap among tests for selection, characterizing population genetic statistics, and characterizing information regarding mapping to the reference genome. We used permutation tests to evaluate whether the proportion of SNPs identified by at least two programs was significantly greater than expected by random chance, and we repeated permutation tests for the proportion of significant SNPs shared by each pair of programs to evaluate consistency in the identification of signatures of selection; each permutation test used 10,000 replicates. We further visually assessed the distributions of global and locality‐specific minor allele frequencies, observed heterozygosity, and missing data for the significant SNPs relative to the entire SNP dataset. We additionally evaluated the distribution of significant SNPs across reference chromosomes.

### 2.7 Additive polygenic scores

Polygenic scores have emerged in the landscape genomics literature as a method to summarize genotype–environment associations across SNPs at the individual level (e.g., Babin et al., 2017; Xuereb et al., 2018), and this implementation is distinct from polygenic scores used in relation to genome‐wide association and quantitative genetics studies that establish links between genetic data and phenotypes and/or fitness (e.g., Wray et al., 2014); accordingly, the use of polygenic scores in the present study concerns their implementation in landscape genomics research and does not involve direct measures of phenotypes or fitness. Additive polygenic scores provide evidence for multilocus or polygenic local adaptation through the co‐occurrence of alleles across multiple significant SNPs within individuals (Gagnaire & Gaggioti, 2016). We created six sets of significant SNPs, one set per environmental variable. The sets contained significant SNPs identified using a GEA test and at least one other GEA or outlier test. Restricting the polygenic scores analysis to SNPs identified by multiple tests for selection may reduce the influence of false positives.

Following Babin et al. (2017) and Xuereb et al. (2018), for each significant SNP, we identified the allele exhibiting a positive association between its frequency and an environmental factor. We then summed, within a given individual, the dosage (0, 1, or 2) of the positively associated allele across all significant SNPs, taking this value as a raw additive polygenic score. We calculated raw additive polygenic scores in R using a modified version of the script provided by Xuereb et al. (2018). Because individuals genotyped using reduced‐representation sequencing can have considerable missing data, we divided the raw additive polygenic score by twice the number of significant SNPs with data present for a given individual. This metric provides a polygenic score as a proportion of the maximum score an individual could theoretically have, given its missing data rate. For each of the six sets of SNPs, we also recalculated polygenic scores in relation to latitude and longitude, instead of the environmental factor. Polygenic scores avoid circularity in the use of significant SNPs found by GEAs in evaluating correlations between polygenic scores and the environment: Given that GEAs are conducted on the basis of allele frequencies at individual SNPs independently, the co‐occurrence of alleles positively associated with the environment across different SNPs within individuals (i.e., the signature of polygenic adaptation) is not necessarily expected.

For the analyses showing a general positive relationship between polygenic scores and the environmental variable across sampling locations, we fitted models to characterize the relationship between percent polygenic scores and each environmental factor. Specifically, we used the function *stan_glmer* from the R package rstanarm (Goodrich et al., 2020) to implement Bayesian Generalized Linear Mixed Models (GLMMs). We specified the binomial family and logit link function to accommodate the [0,1] bounds of our percent polygenic score. We included collection locality as a random effect to account for pseudoreplication. To evaluate the extent to which polygenic scores may be explained by geography (i.e., latitude and longitude), we also fit models containing latitude or longitude as the independent variable, instead of the environmental factor.

We also calculated polygenic scores in relation to each environmental factor using random subsets of the entire SNP dataset. Each random subset contained an identical number of SNPs as the set of SNPs significantly associated with the environmental factor of interest. For each environmental factor, we repeated this process 1000 times and calculated individual percent polygenic scores. Using each replicate, we created a GLMM as described previously. We then compared the beta coefficient for the environmental factor using the observed polygenic scores with the distribution of beta coefficients obtained from the random SNP subsets to evaluate whether the observed coefficient is greater than expected by chance. This provides a basis for comparing the pattern of polygenic scores observed from the significant SNPs to the background genome.

### 2.8 Candidate gene identification

We used the BEDTools *closest* command (Quinlan & Hall, 2010) to identify the *A. mexicanum* gene models nearest each of the significant SNPs found by at least one test for selection. When significant SNPs were near uncharacterized *A. mexicanum* gene models (i.e., those not assigned gene symbols), we searched the mRNA sequences predicted for the gene models against the NCBI Basic Local Alignment Search Tools (BLAST) database (Altschul et al., 1990) using an E‐value threshold of 1e‐5 and retained only the top significant match. We were not able to identify genes near SNPs in RAD loci that mapped to unplaced contigs of the *A. mexicanum* genome during integrated alignment (see *Variant calling and filtering*).

We then searched for Gene Ontology (GO) terms describing the molecular functions, biological processes, and cellular components associated with the candidate genes (Ashburner et al., 2000; The Gene Ontology Consortium, 2019). Our principal source for GO terms and literature related to the candidate genes was Xenbase.org, a biological database specializing on *Xenopus* frogs. We gathered additional information on candidate genes by searching the literature. We carried out GO enrichment analysis on the candidate genes using the PANTHER‐powered system (Mi et al., 2019) on the Gene Ontology Consortium webpage, and we specified the reference organism for GO terms as *Xenopus tropicalis*. We tested for over‐ or under‐representation of biological processes, molecular functions, and cellular components among the candidate genes using Fisher's exact test and an FDR of 0.05 (Benjamini & Hochberg, 1995). We specified the genes nearest all of the SNPs in the dataset as the reference gene set.

## 3 RESULTS

### 3.1 Variant calling and filtering

The de novo RAD locus catalog obtained from Stacks mapped robustly to the *A. mexicanum* genome: 85.0% (2,636,742/3,101,325) of the de novo RAD loci mapped to at least one genomic location. After removing RAD loci with MAPQ < 10 and integrating alignment information with the de novo RAD catalog, 1,317,396 RAD loci remained for further filtering. The subsequent iterative filtering scheme (Table S1) generated a dataset containing 10,527 SNPs and 86 individuals. The central, north, south, and west localities were represented by similar numbers of individuals in the final dataset (23, 20, 20, and 23 individuals, respectively). The dataset had a final mean individual‐level missingness of 19.1% [standard deviation (SD) 13.0%; minimum (min.) 1.8%; maximum (max.) 48.5%], mean SNP‐level missingness of 19.1% (SD 5.4%; min. 2.3%; max. 34.9%), individual read depth averaged across all loci of 9.83 (SD 2.9; min. 6.0; max. 20.9), and an average mean depth per locus across individuals of 10.0 (SD 1.2; min. 7.0; max. 15.7). SNP‐level missingness and individual‐level missingness were generally higher in the southern collection locality, and individual‐level missingness was more variable in the central collection locality (Figure S1A,B). SNP‐ and individual‐level sequencing depth were largely consistent across collection localities (Figure S1C,D). When SNPs were subdivided by the *A. mexicanum* chromosomes they mapped to, the final SNP dataset showed a strong, significant correlation between *A. mexicanum* chromosome size and the number of *A. barbouri* SNPs retained by our pipeline (Pearson's *r* = 0.98, *p *< 10e‐9; Figure S1E), indicating proportional representation of chromosomes. 38% of the 10,527 SNPs in the final dataset mapped within gene models of the reference genome.

### 3.2 Summary statistics and population genetic structure

**FIGURE 2:**
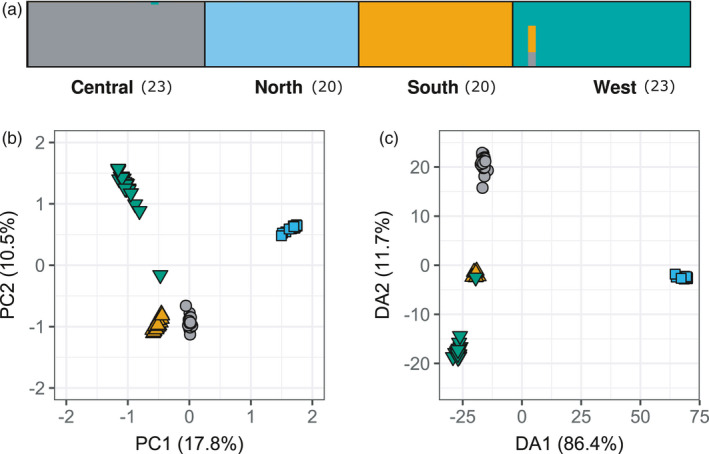
Evidence for population genetic structure among sampling localities. (a) Plot showing fastStructure admixture proportions of individuals for *K* = 4, with individuals grouped by collection locality. Sample sizes are provided in parentheses. (b) Plot of principal component (PC) 2 against PC1. (c) Plot of discriminant axis (DA) 2 against DA1. Individuals in (b) and (c) are colored to reflect sampling locations, using the same colors as in (a). The percentage of variance explained by each PC and DA is provided in parentheses along the axes

FastStructure and the *find*.*clusters* algorithm of Adegenet found the greatest support for four genetic clusters corresponding to the four sampling localities (Figure S2); we therefore grouped individual samples based on collection locality for all subsequent analyses. FastStructure found little evidence for admixture among the four genetic clusters (Figure 2A). Notably, one individual collected from the western locality showed evidence of admixture with the central and southern localities (Figure 2A); this same individual clustered near the central and southern localities in PCA (Figure 2B) and clustered with the southern locality in DAPC (Figure 2C). Genetic clustering revealed by the PCA largely reflects the geographic arrangement of the sampling localities (Figure 2B). PC1 was significantly correlated with both latitude and longitude (latitude: Pearson's *r* = 0.91, *p *< 0.0001; longitude: *r* = 0.89, *p *< 0.0001). PC2 was also significantly correlated with both latitude and longitude (latitude: *r* = 0.28, *p *< 0.01; longitude: *r* = −0.44, *p *< 0.0001. PC1 and PC2 correlated with environmental factors to different extents (Table S4). DAPC highlighted strong isolation between the northern locality and remaining three localities (Figure 2C).

Pairwise *F*_ST_ values were largely consistent with the results of DAPC, as pairwise *F*_ST_ estimates involving the northern population were generally elevated (*F*_ST_ ≥ 0.4225) relative to other pairwise comparisons. Indeed, the highest pairwise *F*_ST_ calculated was between the north and south subpopulations (*F*_ST_ = 0.5185; 95% CI [0.5103, 0.5273]), consistent with these subpopulations being separated by one of the greatest geographic distances between any pair (267 km; see Table S5). The remaining pairwise comparisons further supported strong genetic differentiation across the study area (all pairwise *F*_ST_ values ≥0.2259; Table S5). Recalculation of pairwise *F*_ST_ after removing SNPs determined to have significant signatures of selection resulted in lower estimates of genetic structure (all *F*_ST_ values ≥0.2010) while retaining the same general patterns (Table S5). Environmental distances, geographic distances, and pairwise linearized *F*_ST_ values were all at least moderately correlated (Pearson's coefficient ≥0.45).

The central, northern, and western populations had low estimates of *F*_IS_ at 0.027 [−0.021, 0.019], −0.016 [−0.081, −0.016], and 0.025 [−0.032, 0.29], respectively. The southern population exhibited a higher *F*_IS_ estimate of 0.079 [0.023, 0.071]. Individual observed heterozygosities were lowest in the northern and southern subpopulations, intermediate in the central subpopulation, and highest in the western subpopulation; per‐SNP nucleotide diversity showed a similar pattern (Figure S3). Large proportions of SNPs were invariant within collection localities (central: 37.9%; north: 66.6%; south: 42.0%; west: 26.7%), and 84.9% of SNPs were fixed in at least one collection locality. Point estimates of *N*_e_ were lowest in the western and southern localities, intermediate in the central locality, and highest in the northern locality. However, the upper limit of the 95% CIs included infinity for all localities at an allele frequency threshold of 0.05 and three of four localities at lower allele frequency thresholds (Table S6).

### 3.3 Tests for selection

LFMM 2 identified 512 SNPs significantly associated with at least one of the six environmental factors, and at least 77 SNPs were identified as having a significant association with each environmental factor. Bayenv2 identified 145 SNPs associated with at least one environmental factor. There was some overlap in the SNPs identified by the two GEA analysis programs for a given environmental factor, as well as overlap in the SNPs identified by each program individually across the six environmental factors (Table S7). Among the outlier tests, X^T^X, pcadapt, and FLK identified 200, 354, and zero SNPs, respectively. Collectively, our five tests for selection (i.e., LFMM, Bayenv2, X^T^X, FLK, and pcadapt) identified 732 unique SNPs with signatures of selection deemed significant at the thresholds described above, representing approximately 7.0% of the SNP dataset. Of these significant SNPs, 385 (approximately 52.6%) were identified by at least two tests for selection (Figure S4). As evaluated using a permutation test, this represented significantly greater overlap among tests than expected by random chance (*p *< 0.0001). Overlap among pairs of tests was also generally significantly greater than expected by random chance (*p *< 0.007), bar the overlap for the Bayenv2‐LFMM and Bayenv2‐PCAdapt comparisons (Table S8).

Many SNPs of the total set of 732 significant SNPs were invariant within collection localities (central: 52.7%; north: 60.1%; south: 69.3%; west: 50.1%), and 94.9% were invariant in at least one collection locality. This trend was also apparent among the 385 significant SNPs identified by at least two tests for selection, as many of these SNPs were invariant within collection localities (central: 60.0%; north: 57.1%; south: 76.6%; west: 60.0%), and 96.6% were invariant in at least one collection locality. Distributions of minor allele frequencies, observed heterozygosities, and missing data for the full dataset of 10,527 SNPs, full set of 732 significant SNPs, and the set of 385 significant SNPs found by at least two tests for selection are presented in Figure S5. Significant SNPs were found across all reference chromosomes, even when stratified by the environmental factor they were associated with. The number of significant SNPs found on a given reference chromosome was correlated with chromosome size (Table S9). Numbers of significant SNPs used subsequently in additive polygenic scores analyses and the identification of candidate genes are provided in Table S10.

### 3.4 Additive polygenic scores

**FIGURE 3:**
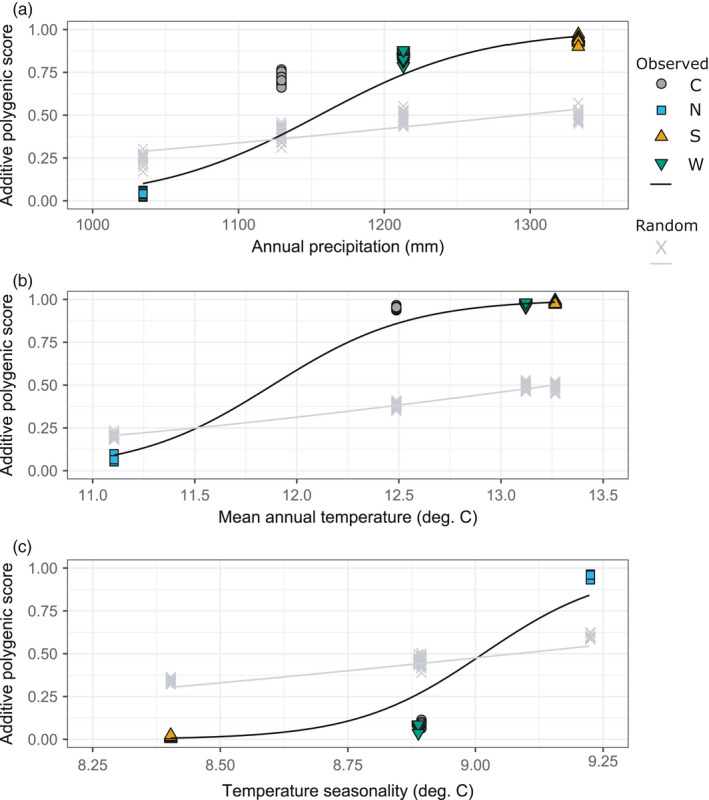
Select polygenic scores. Additive polygenic scores are shown in relation to (a) annual precipitation, (b) mean annual temperature, and (c) temperature seasonality. Polygenic scores were calculated based on SNPs identified as significant in a GEA analysis and at least one other test for selection. Polygenic scores in different panels are based on different but sometimes overlapping sets of SNPs. Scores are reported as a fraction of the maximum possible summed dosage of positively associated alleles an individual could have, given its missing rate. Light gray crosses and curves represent a single representative replicate of polygenic scores calculated from a random subset of the 10,527 SNPs in the full dataset

There was generally limited variation in the additive polygenic scores of individuals within populations (Figure 3A–C). Beta coefficients for annual precipitation, mean annual temperature, and temperature seasonality were positive with 95% Bayesian credible intervals excluding zero (annual precipitation: 0.0177 [0.003, 0.03]; mean annual temperature: 2.976 [1.335, 4.152]; temperature seasonality: 8.188 [1.445, 13.627], indicating a positive relationship between the environmental factor of interest and individual percent polygenic scores. Patterns of polygenic scores at the significant SNPs for all three environmental factors were substantially different from polygenic scores calculated on the basis of random subsets of SNPs (Figure 3A‐C), and permutation tests showed that beta coefficients corresponding to polygenic scores derived from the significant SNP sets were significantly greater than expected by random chance identification of SNPs (*p *< 0.001 for annual precipitation, mean annual temperature, and temperature seasonality). Models containing either latitude or longitude were also fit for each set of polygenic scores. *R^2^* for all models (those including the environmental factor and those including latitude or longitude, along with the random effect of site) were >0.99. For each SNP set, polygenic scores appeared well explained by either the environmental factor of interest or latitude (Figure S6). The relationship between population means of additive polygenic scores and the environment for percent soil organic carbon, elevation, and mean temperature of the wettest quarter were nonmonotonic (Figure S7D–F), and we did not fit models for these variables.

### 3.5 Candidate gene identification

We used BEDtools to identify the *A. mexicanum* gene models nearest each of the 732 significant SNPs. 49 of the 732 significant SNPs occurred in RAD loci that aligned to contigs of the *A. mexicanum* genome assembly that have not been assigned chromosomal positions; we therefore could not identify gene models near these SNPs. All candidate genes were ≤5.82 Mb away from their corresponding significant SNPs (see *Discussion* for commentary on distances), with many SNPs occurring within *A. mexicanum* gene models but not necessarily exons. 130 SNPs corresponded to 123 genes that were uncharacterized or otherwise not assigned gene symbols in the *A. mexicanum* genome assembly; we obtained their predicted mRNA sequences from the UCSC Genome Browser and searched them against the BLAST database. Most of the uncharacterized genes (71/123) did not have significant similarity with BLAST database entries; those that did often had uninformative gene symbols (Table S11).

Many of the candidate genes identified near the significant SNPs are associated with general biological processes (e.g., transcription). We therefore restricted our discussion to several genes that have associations with more specific ecological drivers of local adaptation; a full table of candidate genes, associated GO terms, and additional data can be found in Table S12.

We note WNT7B and AXIN1, which are both related to multicellular organism development (e.g., Zeng et al., 1997), as well as U2AF1L4, which may be involved in regulating the circadian rhythm (Preußner et al., 2014). Among the genes that mapped near SNPs uniquely associated with mean annual temperature, TEX2 has a signature of positive selection in high altitude sheep (Shi et al., 2019) and both ACER1 and PSMG3 are implicated in hypoxia response (Kharrati‐Koopaee et al., 2019; Wang et al., 2013). The genes OTX1 and LVRN were found in relation to both mean annual temperature and temperature seasonality, and they have been implicated in hypoxia response and human altitudinal adaptation, respectively (Yi et al., 2013; Zhang et al., 2020). The MGAT5B gene was near a SNP associated with mean temperature of the wettest quarter, and this gene has previously shown differential expression in association with variation in aquatic habitat permanence (i.e., temporary ponds vs. permanent streams) in larvae of the salamander species *Salamandra infraimmaculata* (Goedbloed et al., 2017). RIN2, found here in association with mean temperature of the wettest quarter, has been implicated in dehydration response (Kordonowy & MacManes, 2017). ZCCHC6 is a hypoxia biomarker (Mosqueira et al., 2012) and was identified here in association with both mean annual temperature and elevation. PIK3R5 has previously been implicated in thermal stress response (Kim et al., 2017) and was found in relation to mean annual temperature and temperature seasonality. Allele frequency shifts for SNPs near these candidate genes are provided in Figure S8. No GO biological process, molecular function, or cellular component terms were significantly over‐ or under‐represented among our list of candidate genes at an FDR of 0.05.

## 4 DISCUSSION

Landscape genomics has emerged as a widely used framework for understanding the spatial distribution of adaptive variation (Joost et al., 2007; Storfer et al., 2018). In light of accelerating climate change, the identification of putatively adaptive variation provides a starting point for assessing whether species may have the capacity to respond to shifting environmental conditions. Using a suite of tests for selection, we identified genomic signatures of local adaptation among populations of *A. barbouri*. These include a subset of RAD sequencing‐derived SNPs showing strong genetic differentiation among populations or strong associations with environmental factors, as well as evidence potentially consistent with polygenic adaptation to several environmental variables. The candidate genes identified near significant SNPs are also plausibly related to known selective pressures in amphibians. Our results suggest that *A. barbouri* may have poor potential to respond naturally to environmental change through *in situ* adaptation or a range shift. Indeed, our results suggest that a range contraction may be more likely. We arrive at this conclusion through a combination of: (1) evidence for local adaptation; (2) evidence for strong range‐wide genetic differentiation, and (3) a synthesis of prior results suggesting that *A. barbouri* conforms to the central‐marginal hypothesis, which we discuss in tandem.

### 4.1 Tests for selection and additive polygenic scores

Approximately 7.0% of SNPs were found to have either significant associations with at least one environmental factor or significant genetic differentiation among populations, indicating that environmental heterogeneity likely plays an important role in shaping spatial patterns of genomic variation across the range of *A. barbouri*.

Additive polygenic scores identify signatures of polygenic adaptation (Gagnaire & Gaggiotti, 2016), and we find a strong association of polygenic scores with annual precipitation (Figure 3A). This suggests that *A. barbouri* populations may be locally adapted to precipitation through a polygenic architecture, although this pattern may also be explained by geography. Indeed, precipitation is extremely important for amphibian larval survival in general, particularly for *A. barbouri* that develop in small ephemeral streams (Petranka, 1998; Sih et al., 2000; Storfer & Sih, 1998).

The northern population has extremely low polygenic scores for mean annual temperature but extremely high polygenic scores for temperature seasonality, relative to the other three populations. The southern, eastern and western populations, taken together, still show a positive relationship with these two environmental variables. The strong differentiation between the north population and the other three populations may facilitate local adaptation (Garcia‐Ramos & Kirkpatrick, 1997; Lenormand, 2002), leading to adaptive fixed differences between the northern population and the other three populations, which are considerably less genetically differentiated from one another. Indeed, the northern population showed evidence of local adaptation, with higher larval survival when reared in northern conditions than core conditions in a reciprocal transplant experiment (Micheletti & Storfer, 2020). Alternatively, it is possible that our GEA analyses may have by chance identified neutral loci exhibiting correlations with environmental factors driven largely by the differentiation of the northern population. Strong population structure and confounding between population structure and the environment likely also contribute to observed patterns of polygenic scores, as polygenic scores can also be explained by geography (i.e., latitude and longitude). Although GEAs account for population structure and our approach to polygenic scores involved the removal of SNPs found by only one test for selection, the influence of false positives may still be substantial (Selmoni et al., 2020), suggesting that future work should focus on validating our results (see Limitations and Future Directions).

In general, the identification of SNPs significantly associated with precipitation and temperature‐related variables, as well as evidence suggestive of polygenic adaptation, supports the importance of these factors as selective pressures on amphibian species. Indeed, amphibians are generally vulnerable to desiccation (e.g., Daszak et al., 2005; McMenamin et al., 2008), and drying of breeding habitat negatively impacts recruitment (McMenamin et al., 2008; Semlitsch & Wilbur, 1988). Other ambystomatids become more active during periods of elevated precipitation (e.g., Trenham, 2001), further suggesting that traits related to desiccation resistance or avoidance could improve fitness in areas that are more water‐limited.

### 4.2 Potential to respond to environmental change

Numerous studies point to species’ geographic distributions changing in response to climate change. For example, many species have begun to track environmental conditions toward the poles (Chen et al., 2011; Hickling et al., 2006; Parmesan et al., 1999; Parmesan & Yohe, 2003). However, this response is contingent upon species having dispersal capabilities sufficient to match the pace of environmental change (Schloss et al., 2012). Taxa with poor mobility, such as salamanders (Smith & Green, 2005), may have poor capacity to respond to environmental change through range shifts (Angert et al., 2011). Indeed, we show very strong genetic differentiation across the geographic range of *A. barbouri*, consistent with prior work (e.g., Storfer, 1999), suggesting that migration rates are low. However, the single individual collected from the western locality that showed evidence for admixture with the central and southern localities suggests that relatively rare, long‐distance dispersal may be possible. Additionally, the northern portion of the geographic range is separated from the majority of the range area by the Ohio River, a large river that putatively acts as a barrier to migration (Micheletti & Storfer, 2017). Resistance to gene flow also becomes more intense toward the range edges (Micheletti & Storfer, 2017), suggesting poor potential for dispersal beyond the current range. As such *A. barbouri* is not expected to track a climate‐induced shift of suitable habitat northward.

Range‐wide, *A. barbouri* largely conforms to the central‐marginal hypothesis, with habitat suitability, genetic diversity, and effective population sizes decreasing along transects from the range core to the range periphery (Micheletti & Storfer, 2015). The present results support previous findings of lower genetic diversity at the extreme southern and northern range edges than at the range core, but the western collection locality appears to have highest genetic diversity of the four localities sampled in our study. However, edge populations studied previously do not universally have lower genetic diversity than the core (Micheletti & Storfer, 2015), so the present results should not be construed as invalidating the general trends identified using substantially more sampling localities in previous research. Reduced genetic diversity may constrain adaptive potential (Gilpin & Soulé, 1986), preventing a species from colonizing new regions with environmental conditions differing from its current distribution (Sexton et al., 2009). Apparent poor dispersal and low genetic diversity at the range periphery together suggest that the range of *A. barbouri* may be unlikely to shift appreciably from its current expanse.

Although some populations of *A. barbouri* may be locally adapted to current environmental conditions (Micheletti & Storfer, 2020), the present evidence for strong genetic differentiation and prior evidence in support of the central‐marginal hypothesis suggest that edge populations may be unable to adapt *in situ* as the environment changes. Low genetic diversity at the range edges (Micheletti & Storfer, 2015) may not only constrain a range shift but also hinder *in situ* adaptation if variation at adaptive loci is limiting. Polygenic scores indicate that the northern population may be strongly locally adapted to relatively low annual precipitation, low mean annual temperature, and high temperature seasonality. Indeed, all individuals sampled from this population have similar polygenic scores (either extremely high or low), indicating the near‐absence of alleles putatively adaptive for alternative environmental conditions. To the extent that the polygenic scores may reflect the effects of selection, the northern population may therefore have a poor capacity to respond to changing environmental conditions through *in situ* allele frequency changes. Migration across the range may also be low enough that the spread of standing adaptive variants may be outpaced by environmental change. Together, potential strong local adaptation, low genetic diversity, and poor migration may contribute to evolutionary mismatch with the environment in future (e.g., Zimova et al., 2016), leading to low population fitness and declining population size in the northern range edge.

Previous studies showed that the southern range edge has the lowest habitat suitability (approximately 15–24%; Micheletti & Storfer, 2015). Larvae from this population also had poor overall survival regardless of rearing conditions in a reciprocal transplant experiment (Micheletti & Storfer, 2020). The elevated *F*_IS_ estimate for the southern population suggests inbreeding as a possible cause. Mean annual temperature is notably higher in this part of the range, and this variable is negatively associated with gene flow (Micheletti & Storfer, 2017), suggesting that warming associated with climate change may result in increasing isolation among populations at the southern range edge and enhanced genetic drift. Taken together, these data suggest maladaptation in this apparent sink population, which may presage loss of the southern range edge. Potential strong local adaptation and genetic isolation in the north, as well as maladaptation in the south, suggest that a range contraction may be likely.

### 4.3 Candidate genes

The recovery of genes involved in development (e.g., AXIN1 and WNT7B) and circadian rhythm (U2AF1L4) may reflect variation in the timing of developmental events, such as metamorphosis. Tests for selection in other species have also recovered genes involved in the circadian rhythm (e.g., Dall’Ara et al., 2016; Geraldes et al., 2014). Indeed, regulation of developmental timing is essential for amphibian species that undergo metamorphosis (Semlitsch et al., 1988; Wilbur & Collins, 1973), and for *A. barbouri* individuals that develop in ephemeral streams.

The repeated identification of hypoxia‐related genes associated with mean annual temperature but not elevation suggests that high temperatures toward the southern area of the range may impose selection on *A. barbouri* through the reduced availability of dissolved oxygen in bodies of water. Numerous genes that mapped near SNPs associated with mean annual temperature have been implicated in adaptation to hypoxia in other species. Research in other ambystomatids has revealed a positive correlation between dissolved oxygen and hatching rate (Sacerdote & King, 2009). Further, time‐to‐hatching and larval development of two ambystomatids were reduced in hypoxic conditions, with survival following hatching also decreasing for one species (Mills & Barnhart, 1999). While it is possible that another variable strongly correlated with mean annual temperature may be the underlying environmental driver for these results, it appears unlikely to be elevation, which has a correlation with mean annual temperature of only *r* = −0.52 across our four study sites (Table S3). That multiple genes identified here have also been identified in other studies of adaptation increases our confidence that they are true positives while also providing evidence for common genetic mechanisms underlying evolutionary responses to environmental variation.

### 4.4 Limitations and future directions

Strong genetic differentiation among subpopulations and confounding between population structure and environmental conditions may increase the false positive rates of various tests for selection (e.g., Frichot et al., 2015; Hoban et al., 2016; Lotterhos & Whitlock, 2015; Novembre & Di Rienzo, 2009; Rellstab et al., 2015). Small numbers of collection localities also limit power to detect genomic signatures of local adaptation and increase false discovery rate (Selmoni et al., 2020). These circumstances are true for our study, and while our decision to calculate polygenic scores using only SNPs found to have significant signatures of selection in multiple tests may serve to limit the influence of false positives, more data are required to better disentangle selection and genetic drift. Given the limitations of our sampling scheme, future research should extend sampling to a larger number of locations across the distribution of *A. barbouri*.

It is also important to note that because our procedure for identifying candidate genes near SNPs used a reference genome assembly of *A. mexicanum*, our set of candidate genes is sensitive to the level of structural conservation between the genomes of *A. barbouri* and *A. mexicanum*. Knowledge of the extent of linkage disequilibrium along the *A. mexicanum* genome is also lacking, although the recombination distance per cM is approximately 5 Mb (Smith et al., 2019). We suspect that recombination between each candidate SNP and variation in or near its corresponding candidate gene may be low, given that distances between them are all ≤5.82 Mb. Given these limitations, the candidate genes identified here should be acknowledged with caution. The generation of additional salamander reference genomes will enable more confident identification of genes involved in adaptation to environmental heterogeneity. This will in turn facilitate management efforts aimed at conserving adaptive potential in the numerous salamander species that are threatened by climate change, land development, and emerging infectious diseases (Blaustein et al., 2010; Collins & Storfer, 2003; Martel et al., 2013; Stuart et al., 2004).

Given that our results are derived from reduced‐representation sequencing, we interrogated only a small fraction of the large genome of *A. barbouri*. Thus, the putatively adaptive genomic variation identified here provides incomplete insight into the genomic architecture underlying local adaptation in this species (Lowry et al., 2017). GEA and outlier tests are also biased toward detecting large‐effect loci, further compounding this limitation (Hoban et al., 2016). Future transcriptomic sequencing or targeted DNA sequencing (e.g., whole‐exome sequencing) may help overcome the difficulties related to the large, repetitive genomes of salamanders and facilitate more complete characterization of adaptive genomic variation (Weisrock et al., 2018).

Additional research should pursue functional validation of candidate loci, which is generally lacking in population and landscape genomics studies (Li et al., 2017; Storfer et al., 2018). Future work could include reciprocal transplant experiments (*sensu* Micheletti & Storfer, 2020) using genotyped individuals to link genomic variation with phenotypic variation and fitness in different environments. Nevertheless, the present study provides an initial characterization of the genomic basis of adaptation across the geographic range of *A. barbouri*, as well as a number of hypotheses concerning the ecological pressures driving adaptation.

### 4.5 Conservation management implications

*A. barbouri* is listed as near‐threatened by the IUCN, owing to its limited geographic range, habitat alteration resulting from human land use, and climate change (Hammerson, 2004). Prior genetic work has provided a baseline understanding of neutral evolutionary processes occurring across the range, including evidence for low genetic connectivity among subpopulations (Micheletti & Storfer, 2015, 2017, 2020; Storfer, 1999). Here, evidence for adaptive differences and strong genetic differentiation among subpopulations, as well as limited genetic diversity within some subpopulations of *A. barbouri* suggests range shifts in response to climate change are unlikely. Consequently, our results could be used as preliminary data for the development of genotyping panels aimed at monitoring neutral genetic variation or a subset of adaptive variation, a method that has been suggested in relation to conservation of other species (e.g., Hohenlohe et al., 2019). Indeed, future management efforts could genotype individuals at neutral SNPs to identify those best suited for genetic rescue of genetically depauperate subpopulations, as well as candidate SNPs to identify those best suited for assisted migration to areas of varying environmental conditions, especially as anthropogenic land use and climate change intensify (Hällfors et al., 2014; Vitt et al., 2010). Considering these management actions is especially pertinent given that *A. barbouri* is an endemic species with a small geographic range; such species are expected to be disproportionately threatened with extinction (Manne & Pimm, 2001; Urban, 2015).

## CONFLICT OF INTEREST

The authors declare no conflict of interest.

## Supporting information

Appendix S1

Appendix S2

## Data Availability

The data that support the findings of this study are openly available in Dryad at https://doi.org/10.5061/dryad.5dv41ns6w.
