# Supplementary material for: Landscape genomics of the streamside salamander: Implications for species management in the face of environmental change
Source: Evol Appl. 2022 Jan 25;15(2):220–36. doi: 10.1111/eva.13321 (PMC8867708; doi:10.1111/eva.13321)
Supplement: Supplementary file 1 — Appendix S1 [file EVA-15-220-s002.docx]

**Appendix 1**

Contents

Table S1. Genotype data filtering scheme.

Table S2. Full set of environmental factors prior to filtering by correlation.

Table S3. Matrix of Pearson’s correlation coefficients for all 25 environmental factors.

Table S4. Correlations between PC scores and environmental factors.

Table S5. Pairwise F*_ST_*, geographic distances, and environmental distances among sampling locations.

Table S6. Effective population size estimates.

Table S7. Numbers of significant SNPs identified by the genetic-environmental association (GEA) analysis software LFMM 2 and Bayenv2.

Table S8. Permutation tests of overlap among SNPs identified by different tests for selection.

Table S9. Chromosomal distribution of significant SNPs.

Table S10. Numbers of significant SNPs used in additive polygenic scores analyses and the identification of candidate genes.

Figure S1. Final dataset missing data rates, sequencing depth, and SNP reference mapping.

Figure S2. Support for different values of K.

Figure S3. Genetic diversity.

Figure S4. Venn diagram indicating numbers of SNPs identified as significant in one or more tests for selection.

Figure S5. Summary statistic distributions of significant SNPs and full dataset.

Figure S6. Additive polygenic scores in relation to environmental factors, latitude and longitude.

Figure S7. Additive polygenic scores.

Figure S8. Allele frequency shifts for SNPs near select candidate genes.

Table S1. Genotype data filtering schemes.

| Step | Filtering mechanism | Value | Individuals remaining | SNPs remaining | Rationale | Software |
| --- | --- | --- | --- | --- | --- | --- |
| 1 | RAD locus MAPQ | 10 | 112 | N/A | Remove RAD loci representing collapsed paralogs | Samtools |
| 2 | Max. heterozygosity | 0.50 | 112 | 2,246,605 | Remove SNPs derived from RAD loci representing collapsed paralogs | Stacks v2.52 |
| 3 | Min. read depth to call a genotype | 5 | 112 | 2,246,605 | Reduce genotype miscalls | VCFtools |
| 4 | Biallelic SNPs | N/A | 112 | 2,246,605 | Compatibility with downstream analyses | VCFtools |
| 5 | Min. minor allele count | 3 | 112 | 946,730 | Remove artefactual SNPs | VCFtools |
| 6 | Population SNP missingness | 0.70 | 112 | 268,623 | Reduce missing data; reduce geographic bias in missing data | VCFtools |
| 7 | Individual missingness | 0.90 | 102 | 268,623 | Reduce missing data | VCFtools |
| 8 | Population SNP missingness | 0.50 | 102 | 134,458 | Reduce missing data; reduce geographic bias in missing data | VCFtools |
| 9 | Individual missingness | 0.70 | 94 | 134,458 | Reduce missing data | VCFtools |
| 10 | Population SNP missingness | 0.40 | 94 | 62,081 | Reduce missing data; reduce geographic bias in missing data | VCFtools |
| 11 | Individual missingness | 0.50 | 86 | 62,081 | Reduce missing data | VCFtools |
| 12 | Minor allele frequency | 0.05 | 86 | 33,709 | Reduce confounding of genetic-environment association tests | VCFtools |
| 13 | One SNP per 100bp | N/A | 86 | 12,713 | Reduce linkage among SNPs | VCFtools |
| 14 | Ɨ Population SNP missingness | 0.40 | 86 | 10,925 | Reduce missing data; reduce geographic bias in missing data | VCFtools |
| 15 | ǂ high mean read depth across individuals | 17 | 86 | 10,527 | Remove SNPs derived from RAD loci representing collapsed paralogs | VCFtools |

Population SNP missingness indicates that a SNP is removed when its missingness is greater than the specified value in at least one of the four collection localities. Individual missingness indicates the maximum proportion of SNPs allowed to be missing within a given individual for that individual to be retained. * Filter equivalent to one SNP per RAD locus. Ɨ Population site missingness filter was repeated because prior removal of individuals with high missing data can alter missingness of SNPs. ǂ SNPs with mean read depth across individuals greater than 3 standard deviations away from the mean (corresponding to a mean read depth of 17) in at least one collection locality were removed.

Table S2. Full set of environmental factors prior to filtering by correlation.

|  | Sampling location | | | |  |
| --- | --- | --- | --- | --- | --- |
| Environmental factor | C | N | S | W | Reference |
| Mean annual temperature (°C) | 12.49 | 11.10 | 13.27 | 13.12 | Fick & Hijmans (2017) |
| Mean diurnal range (°C) | 12.25 | 11.3 | 12.52 | 12.47 | Fick & Hijmans (2017) |
| Isothermality (coefficient of variation) | 34 | 31.42 | 35.95 | 34.47 | Fick & Hijmans (2017) |
| Temperature seasonality (°C) | 8.89 | 9.23 | 8.40 | 8.89 | Fick & Hijmans (2017) |
| Max temperature of the warmest month (°C) | 30.44 | 29.1 | 30.6 | 31.01 | Fick & Hijmans (2017) |
| Min temperature of the coldest month (°C) | -5.57 | -6.87 | -4.22 | -5.15 | Fick & Hijmans (2017) |
| Temperature annual range (°C) | 36.01 | 35.98 | 34.82 | 36.16 | Fick & Hijmans (2017) |
| Mean temperature of the wettest quarter (°C) | 21.07 | 20.09 | 21.40 | 12.90 | Fick & Hijmans (2017) |
| Mean temperature of the driest quarter (°C) | 13.56 | -0.74 | 19.21 | 19.48 | Fick & Hijmans (2017) |
| Mean temperature of the warmest quarter (°C) | 23.13 | 22.11 | 23.38 | 23.73 | Fick & Hijmans (2017) |
| Mean temperature of the coldest quarter (°C) | 1.08 | -0.74 | 2.52 | 1.65 | Fick & Hijmans (2017) |
| Annual precipitation (mm) | 1129.44 | 1034.52 | 1333.03 | 1213.07 | Fick & Hijmans (2017) |
| Precipitation of wettest month (mm) | 118.67 | 116.62 | 130.45 | 120.57 | Fick & Hijmans (2017) |
| Precipitation of driest month (mm) | 71.44 | 63.72 | 80.94 | 76.25 | Fick & Hijmans (2017) |
| Precipitation seasonality (coefficient of variation) | 15.99 | 19.48 | 12.68 | 14.31 | Fick & Hijmans (2017) |
| Precipitation of wettest quarter (mm) | 336.74 | 316.14 | 372.26 | 353.79 | Fick & Hijmans (2017) |
| Precipitation of driest quarter (mm) | 239.59 | 204.79 | 290.03 | 259 | Fick & Hijmans (2017) |
| Precipitation of warmest quarter (mm) | 312.63 | 290.9 | 344.58 | 310.54 | Fick & Hijmans (2017) |
| Precipitation of coldest quarter (mm) | 250.15 | 204.79 | 325.39 | 279.25 | Fick & Hijmans (2017) |
| Elevation (m) | 192.19 | 276.93 | 260.19 | 167.46 | Fick & Hijmans (2017) |
| Solar radiation (kJ m-2 day-1) averaged across months | 14060.34 | 13612.84 | 14365.41 | 14382.94 | Fick & Hijmans (2017) |
| Forest landcover (%) | 51.39 | 42.17 | 72.01 | 76.39 | Homer et al. (2020) |
| Canopy cover (%) | 41.49 | 30.99 | 61.27 | 63.34 | Coulston et al. (2012) |
| Soil pH | 5.50 | 5.80 | 5.34 | 5.32 | Ramcharan et al. (2018) |
| Soil organic carbon (%) | 2.79 | 3.04 | 3.38 | 2.60 | Ramcharan et al. (2018) |

Table S3. Matrix of Pearson’s correlation coefficients for all 25 environmental factors.

| Canopy cover | Solar radiation | Soil organic carbon | Soil pH | Forest habitat | Elevation | Mean temperature of the driest quarter | Mean temperature of the wettest quarter | Temperature annual range | Min temperature of the coldest month | Max temperature of the warmest month | Temperature seasonality | Isothermality | Mean diurnal range | Preciptation of the coldest quarter | Precipitation of the warmest quarter | Precipitation of the driest quarter | Precipitation of the wettest quarter | Precipitation seasonality | Precipitation of the driest month | Precipitation of the wettest month | Annual precipitation | Mean temperature of the coldest quarter | Mean temperature of the warmest quarter | Mean annual temperature |  |
| --- | --- | --- | --- | --- | --- | --- | --- | --- | --- | --- | --- | --- | --- | --- | --- | --- | --- | --- | --- | --- | --- | --- | --- | --- | --- |
| 0.9389 | 0.9938 | -0.0053 | 0.9789 | 0.9161 | -0.5191 | 0.9961 | -0.2946 | -0.4223 | 0.9555 | 0.9562 | -0.8407 | 0.9647 | 0.9903 | 0.9292 | 0.8139 | 0.9408 | 0.9409 | -0.9818 | 0.9685 | 0.723 | 0.9097 | 0.9796 | 0.9638 | 1 | Mean annual temperature |
| 0.9179 | 0.9748 | -0.2691 | 0.9672 | 0.9082 | -0.717 | 0.9812 | -0.498 | -0.1678 | 0.8422 | 0.9966 | -0.666 | 0.8617 | 0.9666 | 0.8032 | 0.6301 | 0.8205 | 0.8283 | -0.8974 | 0.8732 | 0.5202 | 0.7775 | 0.8908 | 1 | 0.9638 | Mean temperature of the warmest quarter |
| 0.9038 | 0.9591 | 0.1904 | 0.9378 | 0.8713 | -0.3507 | 0.9606 | -0.1186 | -0.5908 | 0.9947 | 0.8819 | -0.9322 | 0.9972 | 0.9634 | 0.9772 | 0.9141 | 0.9844 | 0.9776 | -0.9976 | 0.992 | 0.8372 | 0.9623 | 1 | 0.8908 | 0.9796 | Mean temperature of the coldest quarter |
| 0.8986 | 0.8972 | 0.3896 | 0.8912 | 0.8697 | -0.1209 | 0.8693 | -0.0646 | -0.7421 | 0.9813 | 0.7502 | -0.9624 | 0.9604 | 0.8597 | 0.998 | 0.947 | 0.995 | 0.9963 | -0.9705 | 0.9845 | 0.9404 | 1 | 0.9623 | 0.7775 | 0.9097 | Annual precipitation |
| 0.7133 | 0.6943 | 0.6795 | 0.6848 | 0.6759 | 0.215 | 0.6598 | 0.2169 | -0.9251 | 0.8886 | 0.4891 | -0.9622 | 0.8535 | 0.66 | 0.9265 | 0.9624 | 0.9143 | 0.9082 | -0.841 | 0.8705 | 1 | 0.9404 | 0.8372 | 0.5202 | 0.723 | Precipitation of the wettest month |
| 0.9341 | 0.9577 | 0.2333 | 0.9472 | 0.9067 | -0.2912 | 0.9426 | -0.1586 | -0.6253 | 0.9933 | 0.854 | -0.9342 | 0.9849 | 0.9349 | 0.9917 | 0.9139 | 0.9949 | 0.9953 | -0.9974 | 1 | 0.8705 | 0.9845 | 0.992 | 0.8732 | 0.9685 | Precipitation of the driest month |
| -0.9297 | -0.9685 | -0.1832 | -0.9538 | -0.9012 | 0.3476 | -0.9614 | 0.1677 | 0.5855 | -0.9928 | -0.8833 | 0.9236 | -0.9906 | -0.9575 | -0.9822 | -0.9029 | -0.988 | -0.986 | 1 | -0.9974 | -0.841 | -0.9705 | -0.9976 | -0.8974 | -0.9818 | Precipitation seasonality |
| 0.926 | 0.9316 | 0.31 | 0.9256 | 0.8991 | -0.2048 | 0.9072 | -0.1275 | -0.6833 | 0.988 | 0.8033 | -0.9469 | 0.9718 | 0.8964 | 0.9979 | 0.9282 | 0.9975 | 1 | -0.986 | 0.9953 | 0.9082 | 0.9963 | 0.9776 | 0.8283 | 0.9409 | Precipitation of the wettest quarter |
| 0.9023 | 0.9237 | 0.3294 | 0.9109 | 0.8709 | -0.1986 | 0.9074 | -0.0707 | -0.7005 | 0.9955 | 0.8004 | -0.9637 | 0.9833 | 0.9035 | 0.9993 | 0.9479 | 1 | 0.9975 | -0.988 | 0.9949 | 0.9143 | 0.995 | 0.9844 | 0.8205 | 0.9408 | Precipitation of the driest quarter |
| 0.7193 | 0.7676 | 0.5621 | 0.7362 | 0.6717 | 0.035 | 0.7666 | 0.2501 | -0.8567 | 0.9478 | 0.6194 | -0.9985 | 0.9361 | 0.7861 | 0.9505 | 1 | 0.9479 | 0.9282 | -0.9029 | 0.9139 | 0.9624 | 0.947 | 0.9141 | 0.6301 | 0.8139 | Precipitation of the warmest quarter |
| 0.8995 | 0.9132 | 0.356 | 0.9025 | 0.8686 | -0.1667 | 0.893 | -0.0627 | -0.7198 | 0.9916 | 0.7807 | -0.9659 | 0.9762 | 0.8875 | 1 | 0.9505 | 0.9993 | 0.9979 | -0.9822 | 0.9917 | 0.9265 | 0.998 | 0.9772 | 0.8032 | 0.9292 | Preciptation of the coldest quarter |
| 0.8873 | 0.975 | -0.0687 | 0.9493 | 0.8603 | -0.5859 | 0.9952 | -0.2603 | -0.3602 | 0.9311 | 0.9704 | -0.8106 | 0.9523 | 1 | 0.8875 | 0.7861 | 0.9035 | 0.8964 | -0.9575 | 0.9349 | 0.66 | 0.8597 | 0.9634 | 0.9666 | 0.9903 | Mean diurnal range |
| 0.8708 | 0.9364 | 0.2382 | 0.9095 | 0.8338 | -0.311 | 0.9432 | -0.0453 | -0.6276 | 0.9961 | 0.856 | -0.9504 | 1 | 0.9523 | 0.9762 | 0.9361 | 0.9833 | 0.9718 | -0.9906 | 0.9849 | 0.8535 | 0.9604 | 0.9972 | 0.8617 | 0.9647 | Isothermality |
| -0.7558 | -0.7992 | -0.5284 | -0.771 | -0.7107 | 0.0022 | -0.7952 | -0.1974 | 0.8377 | -0.9625 | -0.6534 | 1 | -0.9504 | -0.8106 | -0.9659 | -0.9985 | -0.9637 | -0.9469 | 0.9236 | -0.9342 | -0.9622 | -0.9624 | -0.9322 | -0.666 | -0.8407 | Temperature seasonality |
| 0.884 | 0.9608 | -0.2949 | 0.9462 | 0.8712 | -0.7464 | 0.978 | -0.4656 | -0.1409 | 0.8296 | 1 | -0.6534 | 0.856 | 0.9704 | 0.7807 | 0.6194 | 0.8004 | 0.8033 | -0.8833 | 0.854 | 0.4891 | 0.7502 | 0.8819 | 0.9966 | 0.9562 | Max temperature of the warmest month |
| 0.887 | 0.9319 | 0.2887 | 0.9114 | 0.8522 | -0.253 | 0.9278 | -0.0514 | -0.6696 | 1 | 0.8296 | -0.9625 | 0.9961 | 0.9311 | 0.9916 | 0.9478 | 0.9955 | 0.988 | -0.9928 | 0.9933 | 0.8886 | 0.9813 | 0.9947 | 0.8422 | 0.9555 | Min temperature of the coldest month |
| -0.3969 | -0.3745 | -0.9042 | -0.3574 | -0.3523 | -0.5442 | -0.3442 | -0.5283 | 1 | -0.6696 | -0.1409 | 0.8377 | -0.6276 | -0.3602 | -0.7198 | -0.8567 | -0.7005 | -0.6833 | 0.5855 | -0.6253 | -0.9251 | -0.7421 | -0.5908 | -0.1678 | -0.4223 | Temperature annual range |
| -0.4925 | -0.3913 | 0.7238 | -0.4553 | -0.5481 | 0.6677 | -0.337 | 1 | -0.5283 | -0.0514 | -0.4656 | -0.1974 | -0.0453 | -0.2603 | -0.0627 | 0.2501 | -0.0707 | -0.1275 | 0.1677 | -0.1586 | 0.2169 | -0.0646 | -0.1186 | -0.498 | -0.2946 | Mean temperature of the wettest quarter |
| 0.9257 | 0.9913 | -0.0895 | 0.9749 | 0.9048 | -0.592 | 1 | -0.337 | -0.3442 | 0.9278 | 0.978 | -0.7952 | 0.9432 | 0.9952 | 0.893 | 0.7666 | 0.9074 | 0.9072 | -0.9614 | 0.9426 | 0.6598 | 0.8693 | 0.9606 | 0.9812 | 0.9961 | Mean temperature of the driest quarter |
| -0.4367 | -0.5433 | 0.8444 | -0.5317 | -0.4493 | 1 | -0.592 | 0.6677 | -0.5442 | -0.253 | -0.7464 | 0.0022 | -0.311 | -0.5859 | -0.1667 | 0.035 | -0.1986 | -0.2048 | 0.3476 | -0.2912 | 0.215 | -0.1209 | -0.3507 | -0.717 | -0.5191 | Elevation |
| 0.9975 | 0.952 | -0.0443 | 0.9769 | 1 | -0.4493 | 0.9048 | -0.5481 | -0.3523 | 0.8522 | 0.8712 | -0.7107 | 0.8338 | 0.8603 | 0.8686 | 0.6717 | 0.8709 | 0.8991 | -0.9012 | 0.9067 | 0.6759 | 0.8697 | 0.8713 | 0.9082 | 0.9161 | Forest habitat |
| 0.9861 | 0.9953 | -0.0676 | 1 | 0.9769 | -0.5317 | 0.9749 | -0.4553 | -0.3574 | 0.9114 | 0.9462 | -0.771 | 0.9095 | 0.9493 | 0.9025 | 0.7362 | 0.9109 | 0.9256 | -0.9538 | 0.9472 | 0.6848 | 0.8912 | 0.9378 | 0.9672 | 0.9789 | Soil pH |
| -0.0057 | -0.0555 | 1 | -0.0676 | -0.0443 | 0.8444 | -0.0895 | 0.7238 | -0.9042 | 0.2887 | -0.2949 | -0.5284 | 0.2382 | -0.0687 | 0.356 | 0.5621 | 0.3294 | 0.31 | -0.1832 | 0.2333 | 0.6795 | 0.3896 | 0.1904 | -0.2691 | -0.0053 | Soil organic carbon |
| 0.9673 | 1 | -0.0555 | 0.9953 | 0.952 | -0.5433 | 0.9913 | -0.3913 | -0.3745 | 0.9319 | 0.9608 | -0.7992 | 0.9364 | 0.975 | 0.9132 | 0.7676 | 0.9237 | 0.9316 | -0.9685 | 0.9577 | 0.6943 | 0.8972 | 0.9591 | 0.9748 | 0.9938 | Solar radiation |
| 1 | 0.9673 | -0.0057 | 0.9861 | 0.9975 | -0.4367 | 0.9257 | -0.4925 | -0.3969 | 0.887 | 0.884 | -0.7558 | 0.8708 | 0.8873 | 0.8995 | 0.7193 | 0.9023 | 0.926 | -0.9297 | 0.9341 | 0.7133 | 0.8986 | 0.9038 | 0.9179 | 0.9389 | Canopy cover |

Table S4. Correlations between PC scores and environmental factors.

|  | PC1 | | PC2 | |
| --- | --- | --- | --- | --- |
| Environmental factor | Pearson’s r | p-value | Pearson’s r | p-value |
| Latitude | 0.90908 | 1.09E-33 | 0.278157 | 0.00951 |
| Longitude | 0.885284 | 1.15E-29 | -0.43859 | 2.40E-05 |
| Mean annual temperature | -0.96812 | 2.80E-52 | -0.18996 | 0.079811 |
| Temperature seasonality | 0.671067 | 1.55E-12 | 0.55025 | 4.06E-08 |
| Mean temperature of the wettest quarter | 0.504817 | 7.18E-07 | -0.83232 | 3.09E-23 |
| Soil organic carbon | -0.79123 | 1.25E-19 | -0.29391 | 0.00602 |
| Elevation | 0.263458 | 0.014249 | -0.56256 | 1.73E-08 |
| Annual precipitation | 0.694114 | 1.26E-13 | -0.25661 | 0.017079 |

Table S5. Pairwise *F_ST_*, geographic distances, and environmental distances among sampling locations.

| Sampling location pair | Pairwise *F_ST_* [95% CI]  Prior to removing SNPs significant in GEA and outlier tests | Pairwise *FST* [95% CI]  SNPs significant in GEA and outlier tests removed | Geographic distance (km) | Environmental distance |
| --- | --- | --- | --- | --- |
| C-N | 0.4225 [0.4136, 0.4313] | 0.3402 [0.3331, 0.3477] | 146.79 | 2.58 |
| C-S | 0.2597 [0.2521, 0.2674] | 0.2299 [0.2233, 0.2363] | 122.78 | 3.17 |
| C-W | 0.2383 [0.2324, 0.2438] | 0.2210 [0.2160, 0.2260] | 168.77 | 2.35 |
| N-S | 0.5185 [0.5103, 0.5273] | 0.4361 [0.4282, 0.4444] | 267.41 | 4.18 |
| N-W | 0.4573 [0.4495, 0.4652] | 0.4067 [0.3770, 0.3911] | 295.31 | 4.04 |
| S-W | 0.2259 [0.2198, 0.2319] | 0.2010 [0.1962, 0.2057] | 142.67 | 3.99 |

Environmental distances were obtained from calculations of Euclidean distances among sampling locations following principal components analysis including the six retained environmental factors.

Table S6. Effective population size estimates.

|  | Allele frequency threshold | | |
| --- | --- | --- | --- |
| Locality | 0.05 | 0.02 | 0.01 |
| C | 92.7 [27.6, ∞] | 109.1 [25.2, ∞] | 109.1 [25.2, ∞] |
| N | 124.9 [16.8, ∞] | 175.3 [18.0, ∞] | 175.3 [18.0, ∞] |
| S | 16.4 [4.4, ∞] | 23.5 [6.2, ∞] | 23.5 [6.2, ∞] |
| W | 37.0 [40.3, ∞] | 38.6 [25.8, ∞] | 38.6 [25.8, ∞] |

Table S7. Numbers of significant SNPs identified by the genetic-environmental association (GEA) analysis software LFMM 2 and Bayenv2.

| Software | Mean annual temperature | Temperature seasonality | Mean temperature of the wettest quarter | Precipitation | Elevation | Percent soil organic carbon | Total |
| --- | --- | --- | --- | --- | --- | --- | --- |
| LFMM 2 | 443 | 329 | 77 | 93 | 305 | 155 | 512 |
| Bayenv2 | 18 | 3 | 88 | 2 | 38 | 27 | 145 |
| Total | 461 | 332 | 163 | 95 | 342 | 181 | 654 |

The bottom peripheral cells indicate the total number of significant SNPs found by the two programs for a given environmental factor. The right-hand peripheral cells indicate the total SNPs a program found across all environmental factors. The total number of SNPs across all environmental factors and both programs is provided in the bottom right cell. SNPs sometimes overlapped between environmental factors and/or between the two programs. Accordingly, the totals are not always the sum of adjacent cells.

Table S8. Permutation tests of overlap among significant SNPs identified by different tests for selection.

| Comparison | Proportion of significant SNPs shared | Permutation test p-value |
| --- | --- | --- |
| Shared by ≥ tests | 0.526 | 0.0001* |
| Shared by LFMM and Bayenv2 | 0.005 | 0.9758 |
| Shared by LFMM and XTX | 0.205 | 0.0001* |
| Shared by LFMM and PCAdapt | 0.646 | 0.0001* |
| Shared by Bayenv2 and XTX | 0.036 | 0.0003* |
| Shared by Bayenv2 and PCAdapt | 0.002 | 0.9928 |
| Shared by XTX and PCAdapt | 0.210 | 0.0003* |

* Significant p-value.

Table S9. Chromosomal distribution of significant SNPs.

|  | *Ambystoma mexicanum* reference chromosome | | | | | | | | | | | | | | |  |  |
| --- | --- | --- | --- | --- | --- | --- | --- | --- | --- | --- | --- | --- | --- | --- | --- | --- | --- |
| Significant SNP set | chr1 | chr2 | chr3 | chr4 | chr5 | chr6 | chr7 | chr8 | chr9 | chr10 | chr11 | chr12 | chr13 | chr14 | unplaced contigs | Pearson's r | p-value |
| All | 77 | 72 | 65 | 55 | 50 | 67 | 48 | 48 | 46 | 42 | 45 | 33 | 22 | 13 | 49 | 0.924 | 8.67E-07 |
| ≥2 tests | 42 | 39 | 34 | 23 | 27 | 33 | 27 | 31 | 28 | 19 | 22 | 20 | 13 | 4 | 23 | 0.841 | 8.58E-05 |
| Bio1 | 49 | 43 | 31 | 36 | 34 | 37 | 35 | 28 | 36 | 30 | 25 | 19 | 17 | 5 | 36 | 0.832 | 0.000121 |
| Bio4 | 36 | 35 | 21 | 24 | 19 | 29 | 23 | 24 | 27 | 17 | 20 | 17 | 13 | 4 | 23 | 0.782 | 0.000579 |
| Bio8 | 20 | 16 | 18 | 11 | 15 | 12 | 8 | 11 | 10 | 6 | 9 | 12 | 2 | 2 | 11 | 0.790 | 0.000454 |
| Soil organic carbon | 21 | 13 | 11 | 15 | 16 | 12 | 13 | 19 | 17 | 11 | 10 | 8 | 5 | 2 | 8 | 0.652 | 0.008435 |
| Elevation | 35 | 36 | 24 | 26 | 22 | 27 | 26 | 23 | 29 | 18 | 20 | 18 | 11 | 7 | 20 | 0.819 | 0.000191 |
| Bio12 | 13 | 9 | 9 | 6 | 12 | 7 | 3 | 9 | 7 | 6 | 4 | 6 | 0 | 1 | 3 | 0.749 | 0.001299 |

Bio1, 4, 8, 12 correspond to mean annual temperature, temperature seasonality, mean temperature of the wettest quarter, and annual precipitation, respectively. Pearson’s correlation coefficients and associated p-values reflect the correlation between the number of significant SNPs found on a given reference chromosome and the size of the reference chromosome.

Table S10. Numbers of significant SNPs used in additive polygenic scores analyses and the identification of candidate genes.

| Analysis | Number of significant SNPs |
| --- | --- |
| Polygenic scores (Mean annual temperature) | 317 |
| Polygenic scores (Temperature seasonality) | 296 |
| Polygenic scores (Mean temperature of the wettest quarter) | 84 |
| Polygenic scores (Soil organic carbon) | 155 |
| Polygenic scores (Elevation) | 271 |
| Polygenic scores (Annual precipitation) | 91 |
| Candidate gene identification | 732* |

* All tests for selection collectively identified 732 unique significant SNPs.


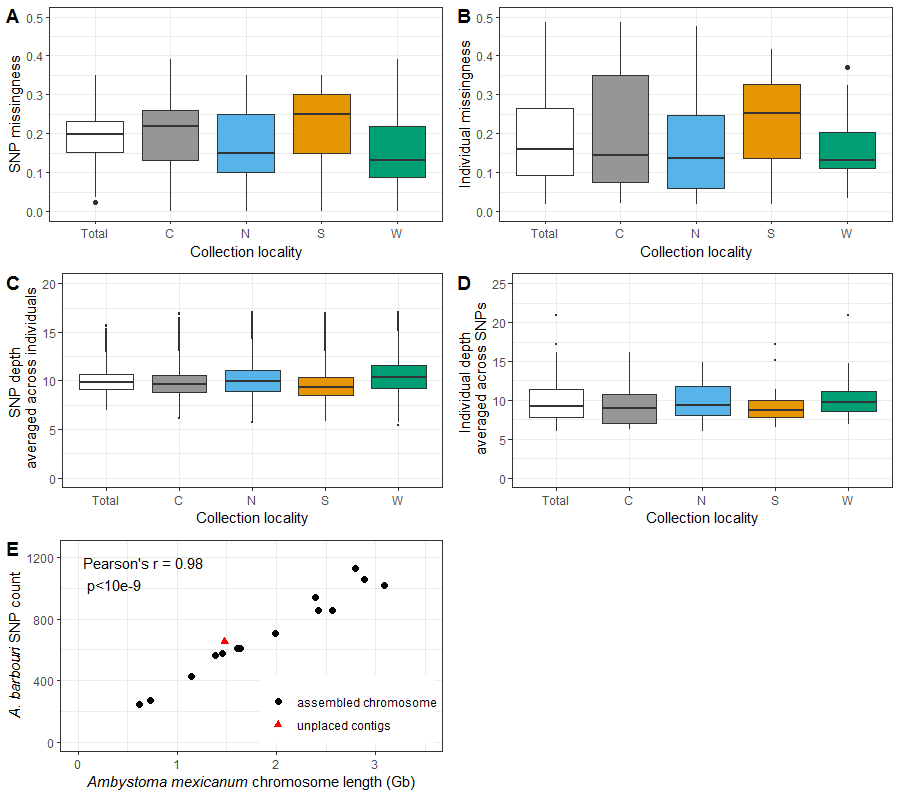


Figure S1. Missing data rates, sequencing depth, and mapping information for the final dataset of 10,527 SNPs. Missing data distributions are provided for A) SNPs and B) individuals. Sequencing depth is provided as C) SNP depth averaged across individuals D) individual depth averaged across SNPs. Missing data rates and sequencing depth are provided for the total set of individuals collectively, as well as on a per-locality basis. E) indicates the correlation between the number of *Ambystoma barbouri* SNPs mapped to a given *A. mexicanum* reference chromosome and the chromosome length. The number of SNPs mapping to unplaced contigs of the A. mexicanum genome are plotted against the total number of base pairs found in unplaced contigs.


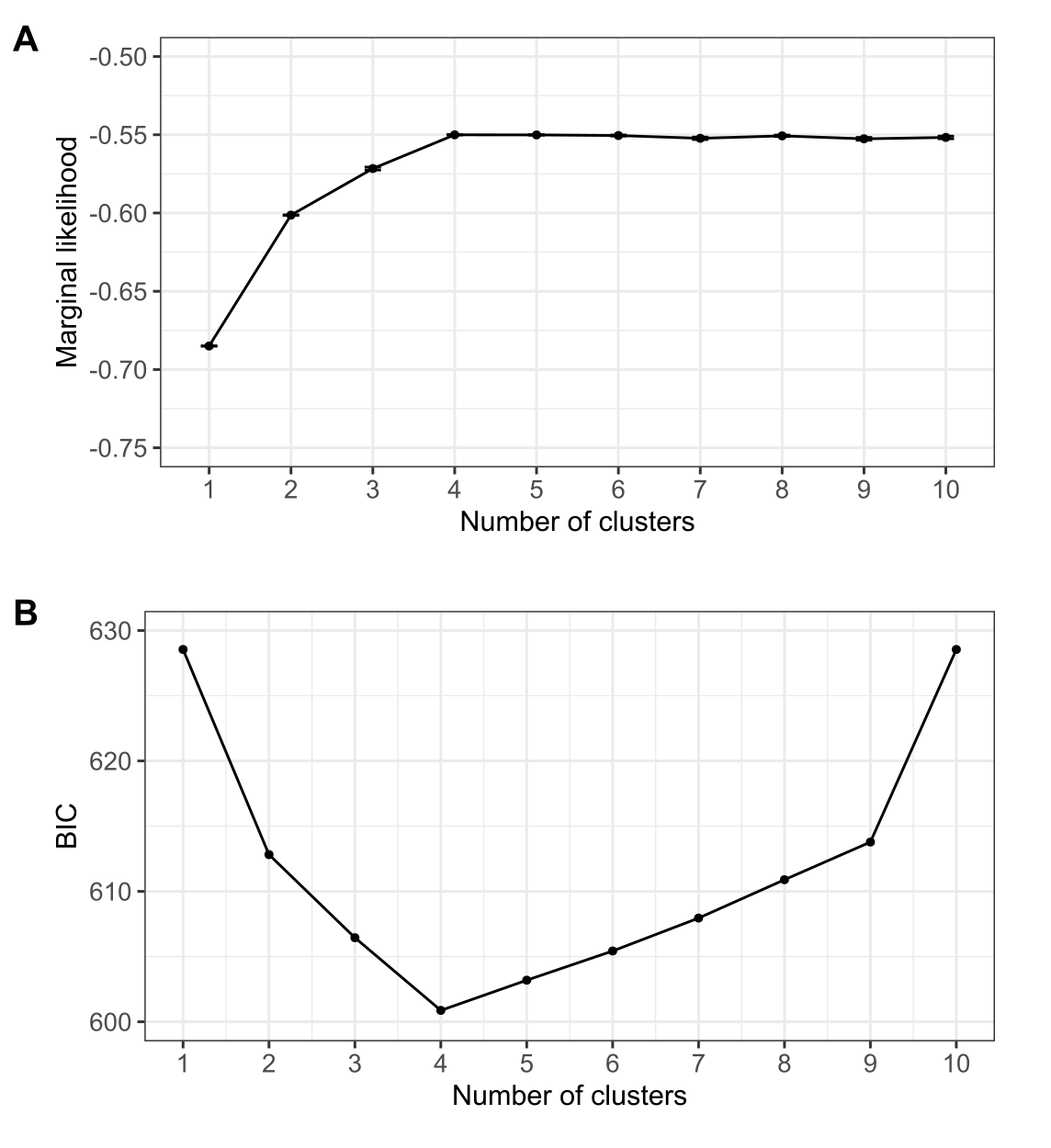


Figure S2. Support for different values of K, as determined by A) fastStructure and B) the find.clusters algorithm of the R package Adegenet.


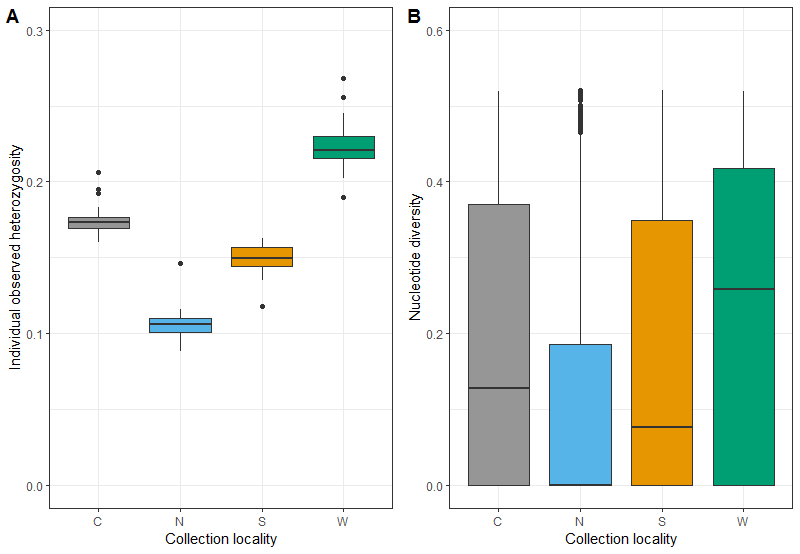


Figure S3. Genetic diversity. Plotted are locality-specific distributions of A) individual observed heterozygosities and B) per-SNP nucleotide diversity (π).


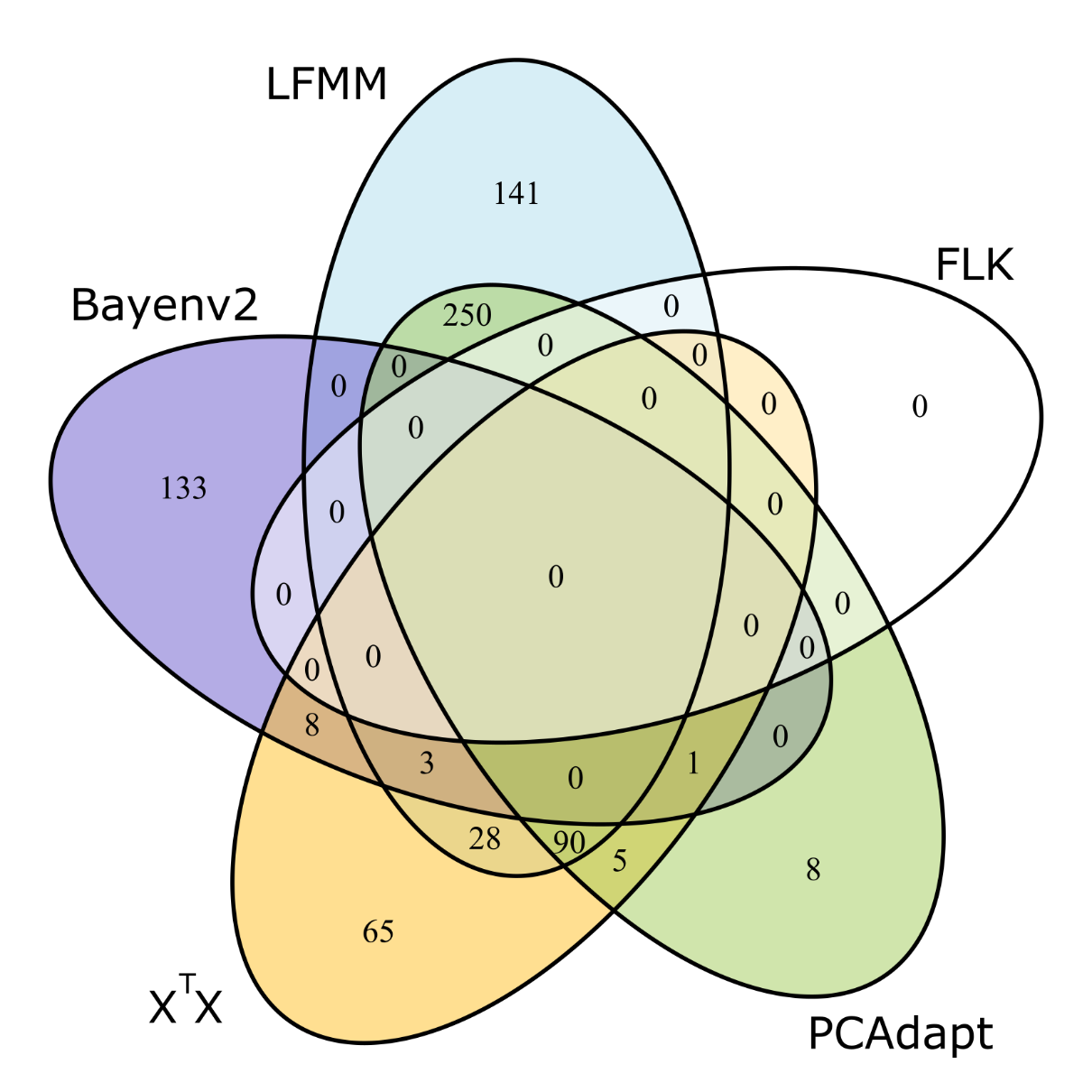


Figure S4. Venn diagram indicating numbers of SNPs identified as significant in one or more tests for selection. The label of Bayenv2 refers to the software’s genetic-environment association test, whereas X^T^X refers to the genetic differentiation test statistic also implemented in Bayenv2.


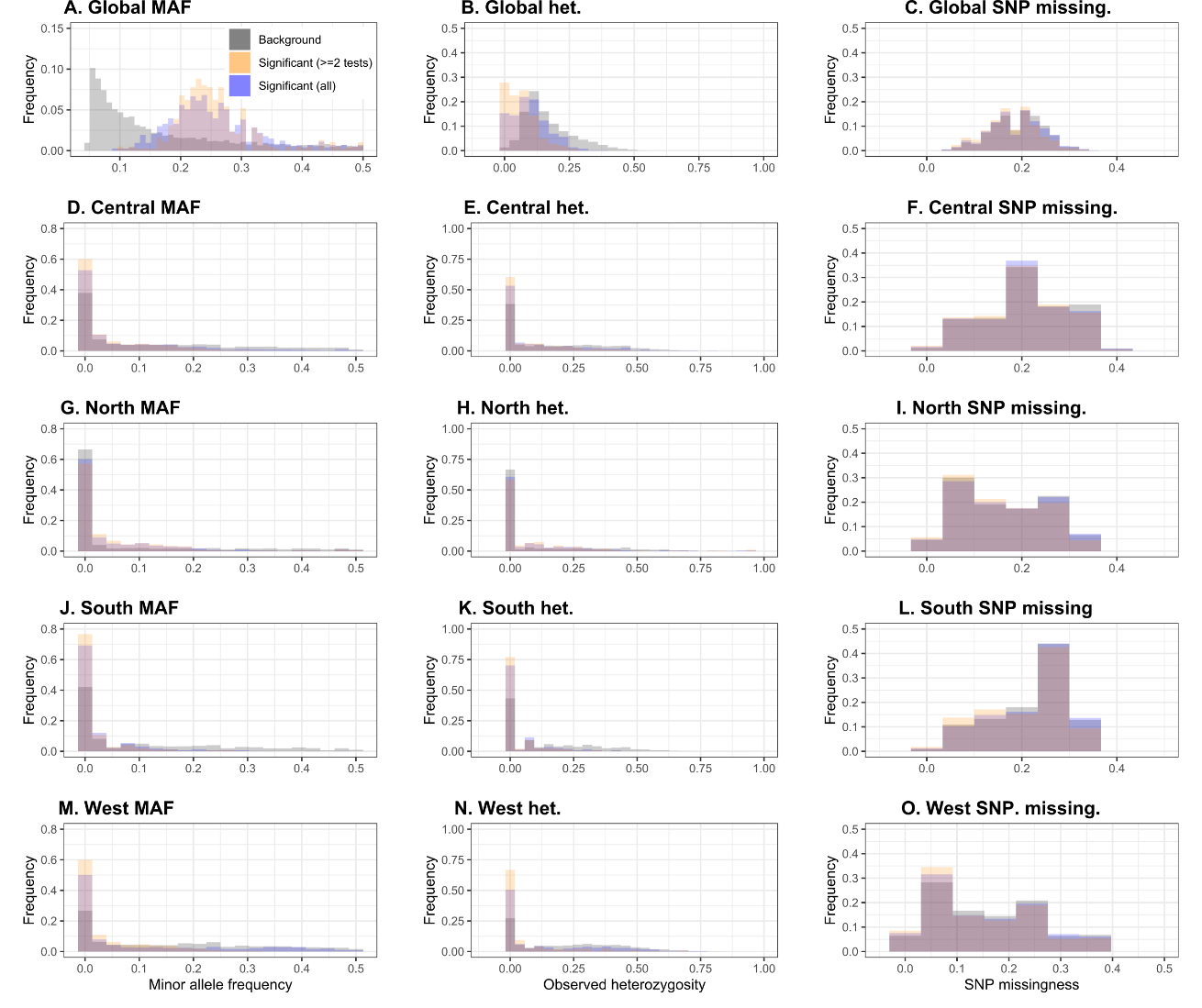


Figure S5. Summary statistic distributions of significant SNPs and full dataset. Minor allele frequency, observed heterozygosity, and missingness of all 10,527 SNPs (gray), significant SNPs in ≥ 2 selection tests (orange), and significant SNPs in ≥ 1 selection test (blue) are plotted for the total (global) set of individuals (A-C), and the central (D-F), north (G-I), south (J-L), and west (M-O) collection localities. Note variation in Y-axis ranges.


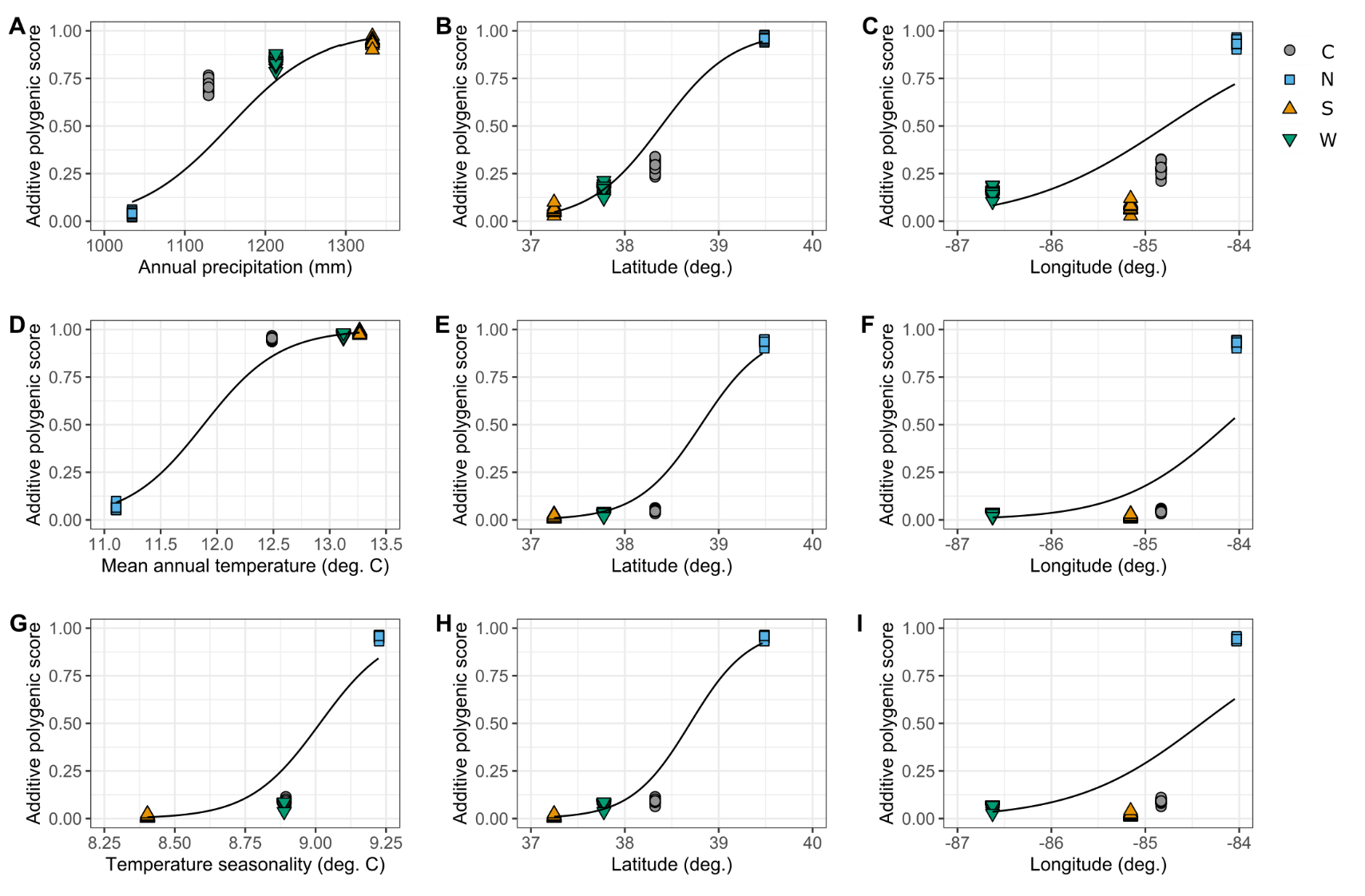


Figure S6. Additive polygenic scores in relation to environmental factors, latitude and longitude. Panels A-C correspond to the SNP set associated with annual precipitation, panels D-F to the SNP set associated with mean annual temperature, and panels G-I to the SNP set associated with temperature seasonality. Polygenic scores were calculated based on SNPs identified as significant in a GEA analysis and at least one other test for selection. Polygenic scores in different panels are based on different but sometimes overlapping sets of SNPs. Scores are reported as a fraction of the maximum possible summed dosage of positively associated alleles an individual could have, given its missing rate. Collection localities are colored as indicated in the key adjacent to panel C.


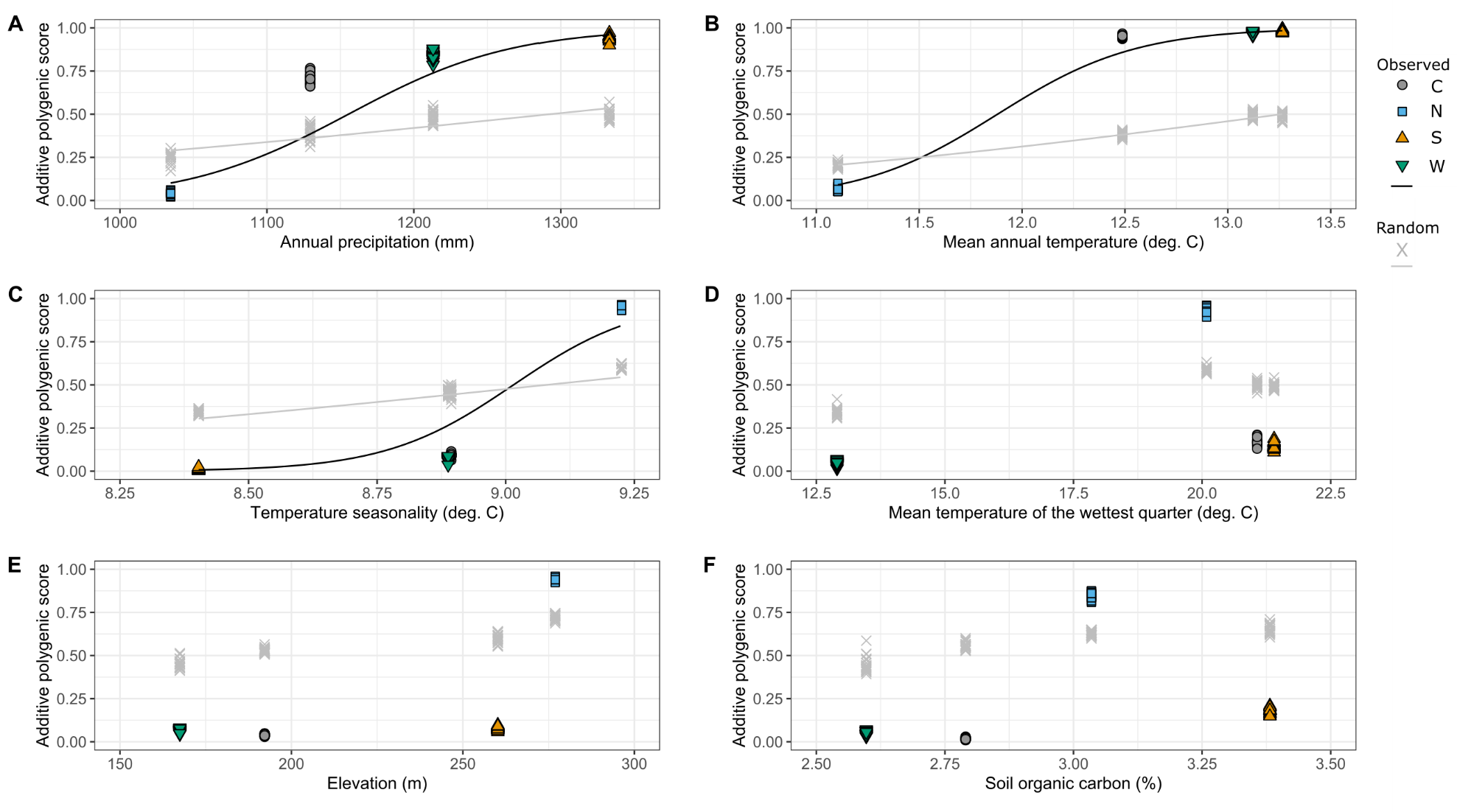


Figure S7. Additive polygenic scores in relation to A) annual precipitation, B) mean annual temperature, C) temperature seasonality, D) mean temperature of the wettest quarter, E) elevation, and F) percent soil organic carbon. Models were not fitted for panels D-F. Polygenic scores were calculated based on SNPs identified as significant in a GEA analysis and at least one other test for selection. Polygenic scores in different panels are based on different but sometimes overlapping sets of SNPs. Scores are reported as a fraction of the maximum possible summed dosage of positively associated alleles an individual could have, given its missing rate. Light gray crosses and curves represent a single representative replicate of polygenic scores calculated from a random subset of the 10,527 SNPs in the full dataset. Collection locality is provided in the key near panel B.


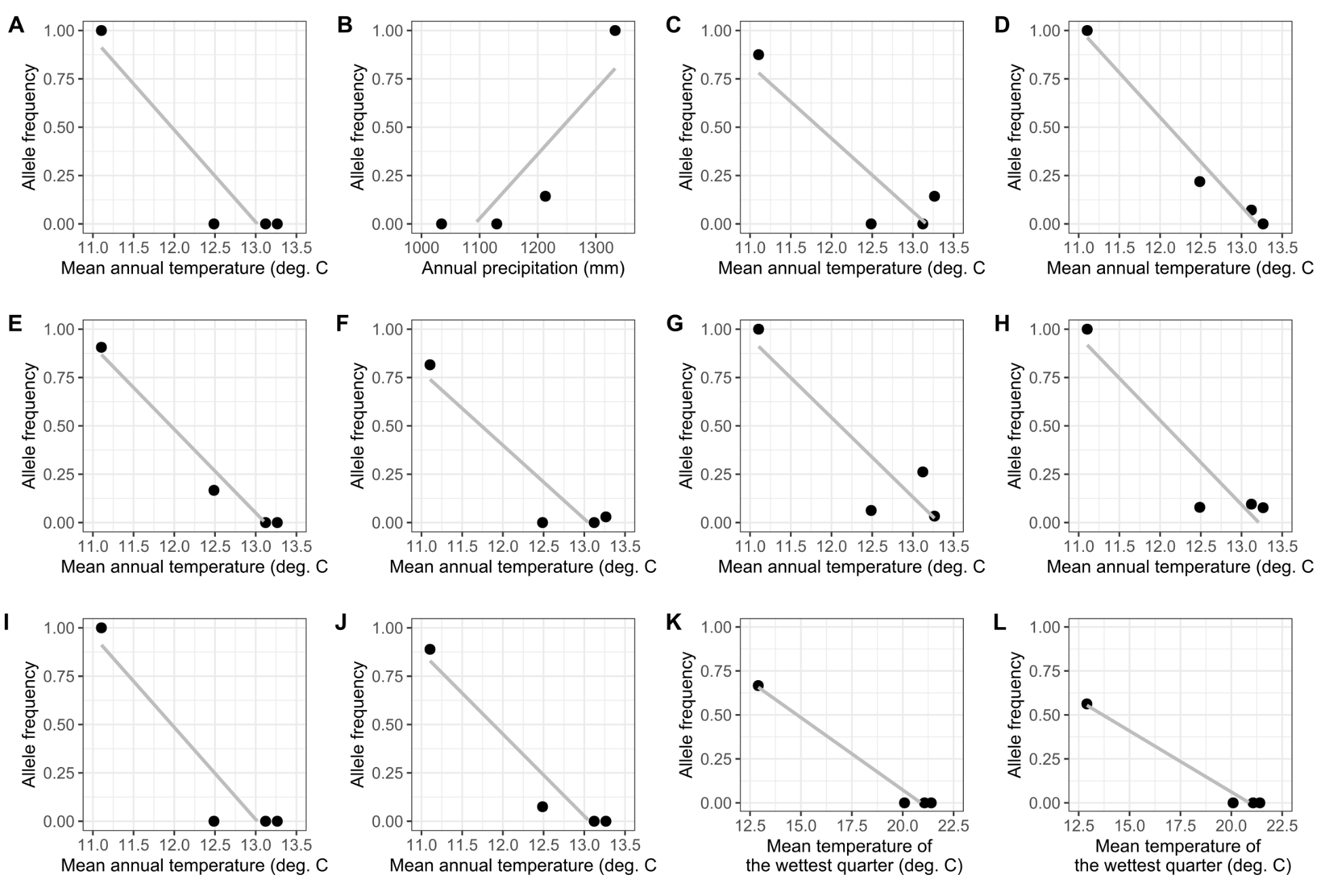


Figure S8. Allele frequency shifts for SNPs near select candidate genes. A) WNT7B, B) AXIN1, C) U2AF1L4, D) TEX2, E) PSMG3, F) ACER1, G) LVRN, H) OTX1, I) ZCCHC6, J) PIK3R5, K) MGAT5B, and L) RIN2. Fitted lines from linear models are shown in gray to aid in visualizing patterns.
